# Structural insights into 3Fe–4S ferredoxins diversity in *M. tuberculosis* highlighted by a first redox complex with P450

**DOI:** 10.3389/fmolb.2022.1100032

**Published:** 2023-01-09

**Authors:** Andrei Gilep, Tatsiana Varaksa, Sergey Bukhdruker, Anton Kavaleuski, Yury Ryzhykau, Sviatlana Smolskaya, Tatsiana Sushko, Kouhei Tsumoto, Irina Grabovec, Ivan Kapranov, Ivan Okhrimenko, Egor Marin, Mikhail Shevtsov, Alexey Mishin, Kirill Kovalev, Alexander Kuklin, Valentin Gordeliy, Leonid Kaluzhskiy, Oksana Gnedenko, Evgeniy Yablokov, Alexis Ivanov, Valentin Borshchevskiy, Natallia Strushkevich

**Affiliations:** ^1^ Institute of Bioorganic Chemistry, National Academy of Sciences of Belarus, Minsk, Belarus; ^2^ Laboratory of Intermolecular Interactions, Institute of Biomedical Chemistry, Moscow, Russia; ^3^ Research Center for Molecular Mechanisms of Aging and Age-Related Diseases, Moscow Institute of Physics and Technology, Dolgoprudny, Russia; ^4^ Frank Laboratory of Neutron Physics, Joint Institute for Nuclear Research, Dubna, Russia; ^5^ Department of Bioengineering, School of Engineering, The University of Tokyo, Tokyo, Japan; ^6^ Institute of Medical Science, The University of Tokyo, Tokyo, Japan; ^7^ European Molecular Biology Laboratory, Hamburg Unit C/O DESY, Hamburg, Germany; ^8^ Institute of Crystallography, University of Aachen (RWTH), Aachen, Germany; ^9^ Skolkovo Institute of Science and Technology, Moscow, Russia

**Keywords:** 3Fe–4S ferredoxins, cytochrome P450, crystal structure, protein–protein interactions, redox complex

## Abstract

Ferredoxins are small iron–sulfur proteins and key players in essential metabolic pathways. Among all types, 3Fe–4S ferredoxins are less studied mostly due to anaerobic requirements. Their complexes with cytochrome P450 redox partners have not been structurally characterized. In the present work, we solved the structures of both 3Fe–4S ferredoxins from *M. tuberculosis*—Fdx alone and the fusion FdxE–CYP143. Our SPR analysis demonstrated a high-affinity binding of FdxE to CYP143. According to SAXS data, the same complex is present in solution. The structure reveals extended multipoint interactions and the shape/charge complementarity of redox partners. Furthermore, FdxE binding induced conformational changes in CYP143 as evident from the solved CYP143 structure alone. The comparison of FdxE–CYP143 and modeled Fdx–CYP51 complexes further revealed the specificity of ferredoxins. Our results illuminate the diversity of electron transfer complexes for the production of different secondary metabolites.

## Introduction

Fe–S proteins such as ferredoxins are ubiquitous and ancient proteins indispensable for life (Beinert et al., 1997). Several lines of evidence suggest that ferredoxins are amongst the oldest proteins on Earth (Hall et al., 1971; Hall et al., 1974). Ferredoxins are responsible for CO_2_ reduction, respiration, and other biological electron transfer (ET) reactions (Braymer et al., 2021).

The diversity of the ferredoxin-containing electron cascades is huge. A wide range of reduction potentials can be achieved with Fe–S clusters of various stoichiometries: [2Fe–2S], [3Fe–4S], [4Fe–4S], [3Fe−4S][4Fe−4S], and 2[4Fe−4S]. Each has their own characteristic Fe–S ligating sequence motif and protein scaffold. Reduction potentials of ferredoxins and interactions with their cognate redox partners could be further tuned by modifying their amino acid sequences (Hosseinzadeh and Lu, 2016; Kim et al., 2016), evolving the protein-controlled, energy-conserving ET pathways. Polyferredoxins composed of three to seven 2[4Fe–4S] modules have also been reported (Watanabe and Shima, 2021).

The [4Fe–4S] clusters are considered the first to have evolved (Beinert et al., 1997; Meyer, 2008) and ferredoxins of this type are the most ubiquitous and abundantly present in anaerobic organisms, whereas [2Fe–2S] cluster-type ferredoxins are abundant in aerobic organisms (Nzuza et al., 2021). The [3Fe−4S] cluster can be considered as a cubane [4Fe−4S] cluster missing one of the irons (Campbell et al., 2019). This class of ferredoxins is found exclusively in bacteria. The [3Fe−4S] clusters can emerge from oxidative damage of [4Fe−4S] clusters; it has been hypothesized as an adaptation to the increased oxygen concentration (Tilley et al., 2001). Some [4Fe–4S] clusters that contain a Cys-X-X-Asp-X-X-Cys motif can undergo reversible cluster interconversion to [3Fe–4S] (Gao-Sheridan et al., 1998), while most ferredoxins containing [3Fe–4S] clusters do not demonstrate such ability. The factors that control assembly and conversion of these clusters are unknown.

More complex 2[4Fe–4S] clusters are proposed to emerge from the gene duplication (Meyer, 2008).

The explosive data from genome sequencing and metagenome analysis have revealed that bacterial genomes often have a larger number of genes that encode multiple ferredoxins. However, the experimental data on their function and redox partners are largely missing. For example, the reduced genome of *M. tuberculosis* (Mtb) *H37Rv* encodes five ferredoxins, Fdx (*Rv0763c*), FdxA (*Rv 2007c*), FdxC (*Rv1177*), FdxD (*Rv3503c*), and FdxE (*Rv1786*). In addition, it also contains two ferredoxins within fusions, FprB (*Rv0886*) and FdxB (*Rv3554*). In FprB, the 4Fe–4S ferredoxin domain is arranged at the N-terminus upstream of the reductase domain, while FdxB additionally has a fatty acid desaturase domain at the N-terminus, followed by the ferredoxin reductase domain and a C-terminal 2Fe–2S ferredoxin domain. The function and/or redox partners of these fusions are currently unknown. Such diversity might serve many different physiological functions yet to be defined.

Interestingly, two Mtb 3Fe–4S ferredoxins, Fdx and FdxE, are co-located in respective operons with cytochrome P450 (CYP) enzymes suggesting their functional relevance. CYPs catalyze the production of primary and secondary metabolites and are involved in the oxidation of a vast range of environmental toxins and drugs. For catalysis, CYPs required electrons typically supplied by redox proteins, such as ferredoxins. Growing evidence has demonstrated that redox proteins can affect the catalytic rate, product profile, and the type and selectivity of P450-catalyzed reactions under varied environmental and cellular conditions (Li et al., 2020). Recent comparative analysis of secondary metabolite biosynthetic gene clusters, ferredoxins, and CYPs in Bacteroidetes and Firmicutes species from human gastrointestinal microbiota indicates that these two bacterial groups produce different secondary metabolites (Nkosi et al., 2022), which might correlate with their contrasting effects on human health. However, such systematic analysis is not available for the species belonging to actinomycetes or to the genus *Mycobacterium* in particular.

It is known that non-sterol-producing Mtb Fdx is capable of supporting CYP51B1 (*Rv0764c*) sterol demethylase activity functioning within the redox chain FprA−Fdx−CYP51B1 (Zanno et al., 2005; McLean et al., 2006). The ferredoxin *Rv1786* gene is adjacent to CYP143 (*Rv1785c*) whose function is not defined. The binding affinity measured for the pair FdxE–CYP143A1 suggests that they form a redox complex (Lu et al., 2017). However, structural details of redox complexes formed by bacterial ferredoxins remain largely unexplored, hampering the understanding of directional ET from/to cognate ferredoxin partners.

Here, we explored the genome context for two ferredoxins, Fdx and FdxE, determined the binding kinetics between FdxE and CYP143, and solved three crystal structures: Fdx, CYP143, and a complex FdxE–CYP143. The complex Fdx–CYP143 represents the first of its kind and we additionally complemented our structural studies by SAXS data in solution. Overall, our experimental work, along with AlphaFold2 predictions of an additional relevant redox complex, shed light on the specificity of the [3Fe–4S] cluster-containing ferredoxins. Exploring the variety and selectivity of electron carriers in biological processes is crucial for their potential applications as biosensors, biofuel cells, pharmaceuticals, and in catalysis.

## Results

### Crystal structure of Fdx (Rv0763c)

The ferredoxin Fdx was the first among the ferredoxins discovered in Mtb and since then has served as an auxiliary redox partner for all known by that time CYPs. Soon after Mtb genome sequencing, CYP51 gene function was established and its crystal structure was solved (Bellamine et al., 1999; Podust et al., 2001). An unusual for mycobacteria steroid demethylase activity of CYP51 was reconstituted with Fdx (Zanno et al., 2005; McLean et al., 2006), confirming their redox partnership. Recent progress highlighted a set of different types of ferredoxins in Mtb*,* but linking them to their cognate redox partners is far from completion.

**FIGURE 1:**
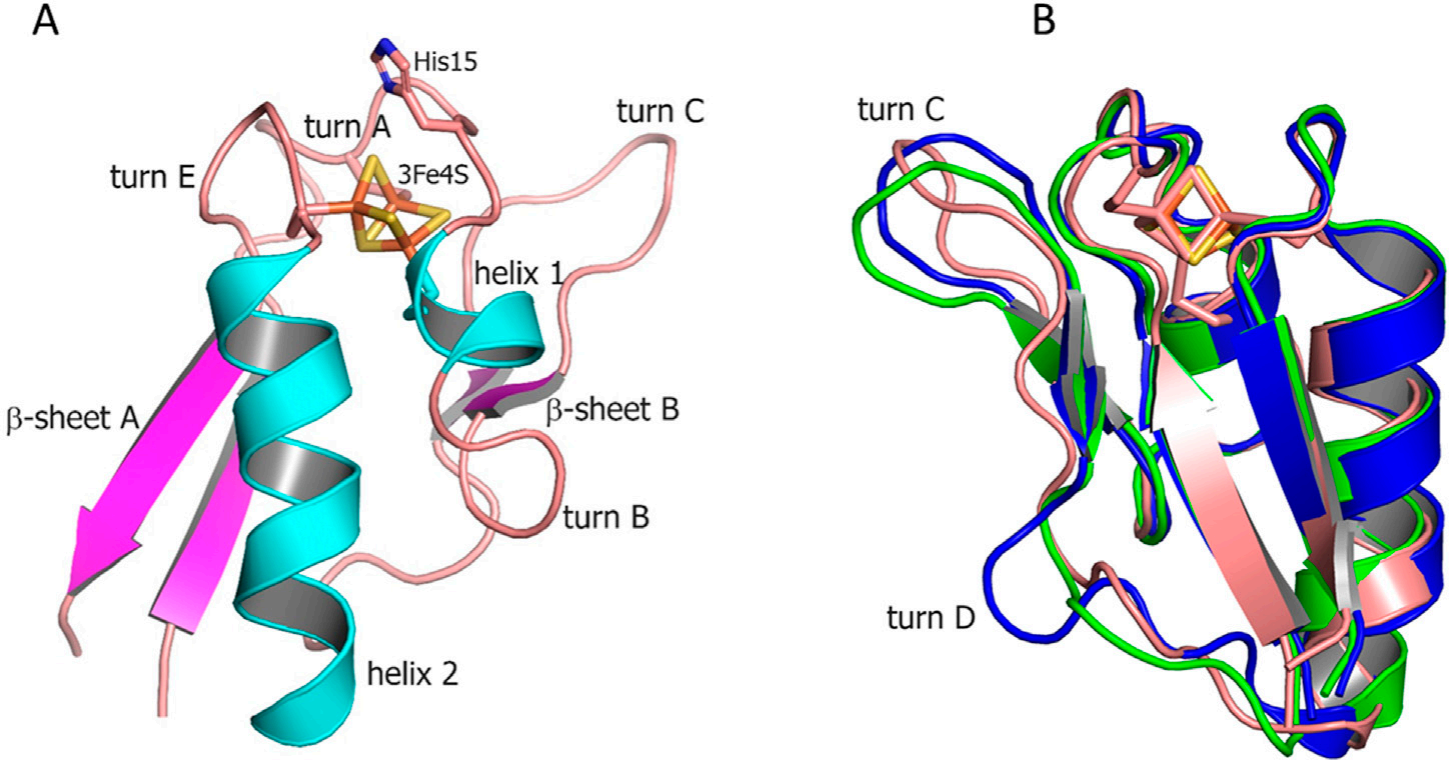
**(A)** Overall structure of Fdx ferredoxin of *M. tuberculosis*. **(B)** Structural alignment of Fdx (salmon) with other structurally characterized 3Fe–4S ferredoxins from *R. palustris* HaA2 (PDB: 4OV1; blue) and *P. furiosus* (PDB: 1SJ1; green). The Fe–S clusters are shown in stick representation (Fe, orange; S, gold).

To obtain a structural insight into a single 3Fe–4S type of ferredoxin, we determined a crystal structure of Fdx at 2 Å resolution (see Supplementary Table S2). The observed overall fold is typical for the monocluster ferredoxins with two double-stranded antiparallel β-sheets and two α-helices. A short α-helix 1 (Met17–Glu21), a longer α-helix 2 (Glu43–Ala55) and two antiparallel β-sheets, a longer β-sheet A (Tyr3–Ala7 and Leu61–Glu65), and a short β-sheet B (Phe26–Agr27 and Glu35–Ile36) are well-defined (Figure 1A). The structure contains four reverse turns: A, B, C, and E. Turn D is not evident in Fdx forming a loop. Turn C (residues 29–33) forms a rather noticeable protrusion from the surface of the protein. The protruding turn is adjacent to the iron–sulfur cluster and considered to be important in specific interactions with ET partners (Kissinger et al., 1991) (discussed as follows).

The [3Fe–4S] cluster is bound by cysteine residues 12, 18, and 56. The protein contains no cysteines other than those directly involved in cluster binding. A single [3Fe–4S] cluster geometry is shown in Supplementary Figure S1 including values of individual bond lengths and angles for both Mtb ferredoxins (FdxE discussed as follows). None of the average values for corresponding bonds differs significantly between two solved structures and when compared with other 3Fe–4S ferredoxins. The values for S–Fe–S angles, however, differ between ferredoxins.

**FIGURE 2:**
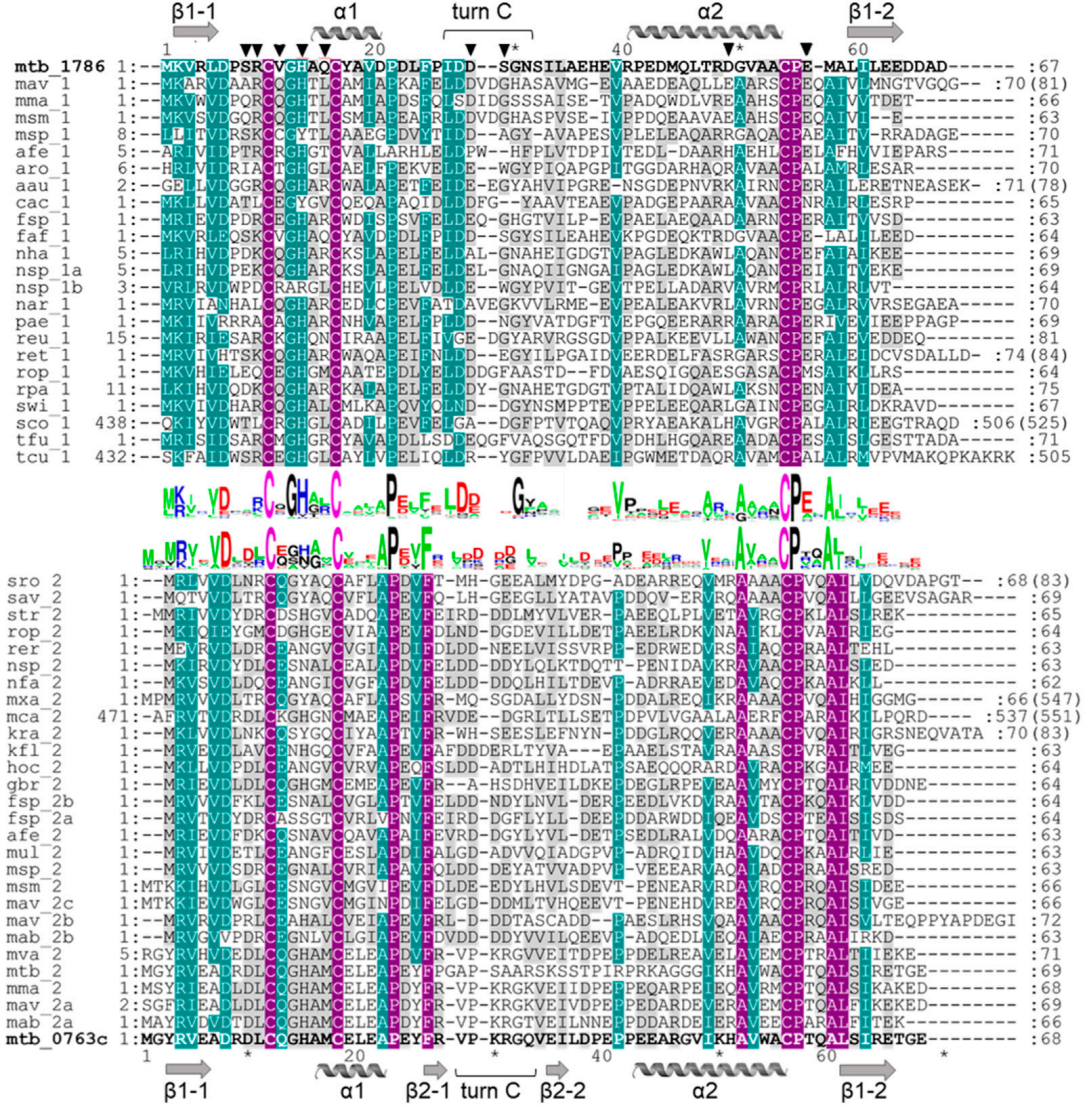
Multiple sequence alignment of ferredoxins taken from the genome context of *Rv1786* (mtb_1786, NP_216302.1) and *Rv0763c* (mtb_0763c, WP_003403890.1) of Mtb (Supplementary Figure S3). Description for abbreviation of proteins is given in Supplementary Figure S2. Alignment was prepared using ClustalW algorithm. Sequence logos were prepared using WebLogo (https://weblogo.berkeley.edu/). Violet color indicates identical residues, turquoise color—residues with 75% of homology, and gray color—residues with 35% of homology. Numbers at the beginning and end of each row correspond to the first and the last amino acids of each sequence taken for alignment, respectively. Numbers in brackets indicate the total number of amino acids in the corresponding proteins. Amino acids important for the interaction with redox partner are shown as triangles.

Both Fdx and FdxE have the same CXXHXXC(X)nCP motif, where proline is invariantly conserved across all 3Fe–4S ferredoxins (Figure 2). The His15 residue does not form a hydrogen bond to S2 of the [3Fe–4S] cluster (distance of 3.6 Å); its side chain is turned away (Figure 1A).

From the sequence alignment, it appears that turn C amino acid composition is specific for Fdx and a small group of related ferredoxins (Figure 2). Indeed, when the Fdx structure is superimposed to other ferredoxins, turn C differs markedly (Figure 1B). Overall, ferredoxins deduced from the genome context of both Fdx and FdxE (Supplementary Figure S3) can be divided to two groups: one is more widely distributed and has classical Asx type (where an additional hydrogen bond is present between a carbonyl oxygen atom of the side-chain of residue n and the amide group of residue n+3) of turn C, while the second group is similar to the Fdx of Mtb and less represented. Furthermore, the FdxE group species without the His residue in the motif do not have CYP genes in the immediate vicinity. However, in the case of Fdx homologs, His might be substituted with N/Y/S (*M. avium, M. abscessus, Haliangium ochraceum, Ktenobacter racemifer* etc), but in the gene surrounding it is always CYP. Based on this observation we could suggest that His is not indicative of a redox partner. Further studies will be required to make any correlations.

Homologs of *Rv1786* are present in other species, e.g., in *Frankia sp, Thermobifida fusca* (both have 43% of homology), *Streptomyces griseus* (40%), *Rhodococcus*, *Nocardioides* (39%), *Rhizobium etli* (34%), *Rhodopseudomonas palustris* (36%), *Arthrobacter aurescens* (33%), *Conexibacter woesei* (34%), *Thermosipho africanus* (33%), and *Sphingomonas* sp. (32%) (Dehal et al., 2010). In most cases, the *Rv1786* gene is organized in one operon with respective CYP (Supplementary Figure S3A), which might be an evolutionary advantage; however, its function is not yet defined. Exceptions, where *Rv1786* homologs are present alone, could also be found (e.g., *Mycobacterium avium* and *Rhodococcus opacus*), but from their different gene environments, it is hard to deduce a common function. In Mtb, CYP143 and *Rv1786* genes are organized within the ESX-5 type VII secretion system implicated in the virulence (Groschel et al., 2016). Despite recent advances in the structural characterization of the ESX-5, the specific substrates translocated by this system are still not identified (Beckham et al., 2017; Bunduc et al., 2021).

### Interactions between FdxE and CYP143

We used an SPR analysis for direct monitoring of the interaction between FdxE and CYP143. Biotinylated CYP143 was specifically immobilized on a streptavidin-coated chip, while FdxE was used as an analyte. This immobilization strategy ensures that all molecules are in the same orientation and a high protein density is achieved not affecting the potentially interacting sites. Analysis of the kinetic parameters of the complex formation between CYP143 and FdxE (Table 1 and Supplementary Figure S4) demonstrated high-affinity binding with a K_d_ value in the nanomolar range (84 nM). The FdxE−CYP143 complex is characterized by a high on-rate (k_on_ ∼ 10^5^ M^−1^s^−1^) and high off-rate constants indicating a specific yet transient interaction. The results are in accordance with the biological role of ferredoxins as an electron carrier that shuttle electrons between CYP and reductase. Notably, obtained kinetic constants characterizing the complex formation significantly differ from those previously published (Lu et al., 2017). In our experiment, the FdxE−CYP143 complex was formed (k_on_) and dissociated (k_off_) more than 210 and 52 times faster, respectively, than reported (Lu et al., 2017), which could be explained by different immobilization strategies and experimental conditions. The main contribution to the high affinity of CYP143 for FdxE is the high rate of complex formation. Such a rate of complex formation is comparable with that for antibodies, indicating that the interaction is in diffusion-limited mode (Schreiber et al., 2009). To reduce the contribution of the diffusion effect, we used a relatively high flow rate in our experiments.

**TABLE 1:** Kinetic and thermodynamic parameters of the complex formation between FdxE and CYP143.

k_*on*_, 10^5^ M^−1^s ^−1^	k_*off*_, s^−1^	τ_1/2_, s	K_d_, nM	ΔG, kJ/mol	ΔH, kJ/mol
5.02 ± .07	.0423 ± .0005	16.4 ± 0.2	84 ± 2	−40 ± 8	−45 ± 9

Analysis of the obtained thermodynamic parameters (Table 1) shows that the FdxE−CYP143 complex is enthalpy-driven (ΔH<0 value), suggesting that the main contributions to complex stabilization are electrostatic interactions and hydrogen bonds (Stites, 1997). The positive value of the entropy component (−TΔS>0) may indicate the desolvation of polar and charged amino acids upon complex formation and/or the solvent release from the inter-protein region (Brady and Sharp, 1997).

### Overall structure of the FdxE−CYP143 complex

**FIGURE 3:**
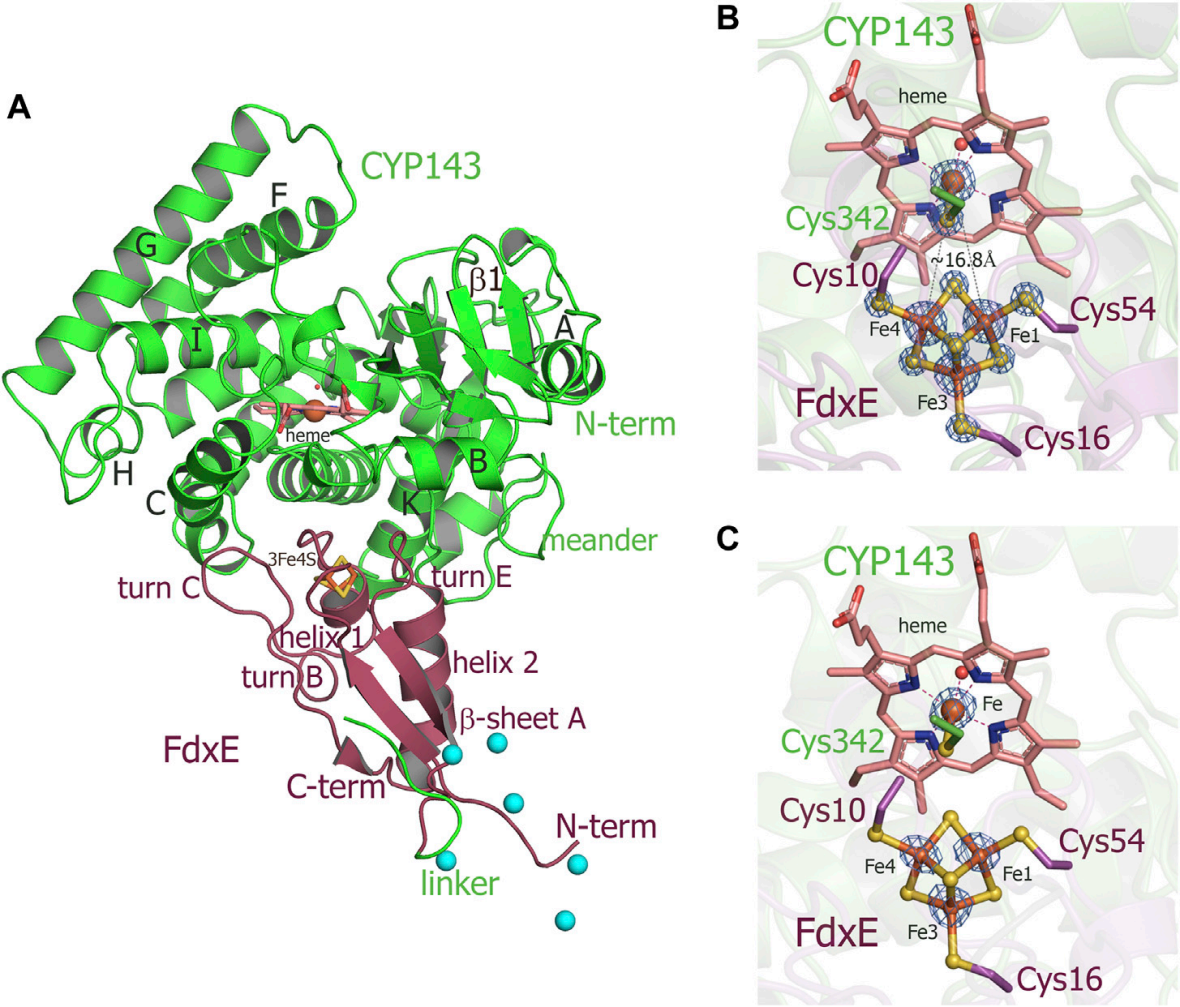
**(A)** Overall structure of the FdxE–CYP143 fusion protein. **(B,C)** Zoomed-in view of redox cofactors. **(B)** 2Fo-Fc maps are countered at 7σ, donor–acceptor edge to the edge distance is shown as gray dashed lines. **(C)** Anomalous difference Fourier maps contoured at 4 σ. FdxE binds (raspberry) at the proximal face of CYP143 (green). Cyan spheres are Ni ions from crystallization conditions bound to His-tag. The N-term His-tag of the FdxE introduced to facilitate purification by metal affinity chromatography is visible and chelated by the Ni ions from crystallization conditions, while the electron density for the part of the linker between two proteins and the first eight residues of CYP143 cannot be resolved.

Taking into account experimentally determined both high off- and on-rates and low stability of the complex (τ_1/2_ = 16.4 s), it seems challenging to trap this transient interaction for structural studies. Here, we applied a fusion strategy proven useful in our previous structural studies of the complex between human adrenodoxin and CYP11A1 (Strushkevich et al., 2011). We constructed a single protein containing FdxE fused *via* a linker to the N-term of CYP143. This protein was used to obtain a crystal structure of the FdxE−CYP143 complex at 1.6 Å resolution as well as for SAXS studies in solution. In the structure, FdxE binds to the proximal surface of CYP143 and has a typical fold of monocluster ferredoxins as described previously for Fdx. CYP143 fold is characteristic for cytochrome P450 and without the ligand presented in the open conformation. The water molecule coordinates heme iron (Figure 3). The protein–protein interface is well-defined.

The buried area upon the complex formation between FdxE and CYP is 1,809 Å^2^. The shortest distance between the CYP143 heme group and the ferredoxin iron–sulfur cluster is 16.75 Å. As expected, several structural regions from the CYP143 proximal side participate in a complex formation, namely, helices C, K, and L and the meander preceding the heme-containing loop. However, some specifics of the 3Fe–4S ferredoxin−CYP complex can be identified. First, the angle of FdxE approaching CYP, second, a more extended interaction interface (additionally involving the B helix of CYP and turn С of FdxE), and third, the shape/steric complementarity. Of the latter, particularly interesting is the complementarity between helix 2 of FdxE and CYP meander running along each other; between helix 1 of FdxE and K helix of CYP; a positioning of the heme coordinating loop right above the [3Fe–4S] cluster, and finally, a perfect fit of turn C of FdxE between С and D helices of CYP. Altogether, it indicates a high specificity of this type of ET complex.

### Interactions between FdxE and CYP143

**FIGURE 4:**
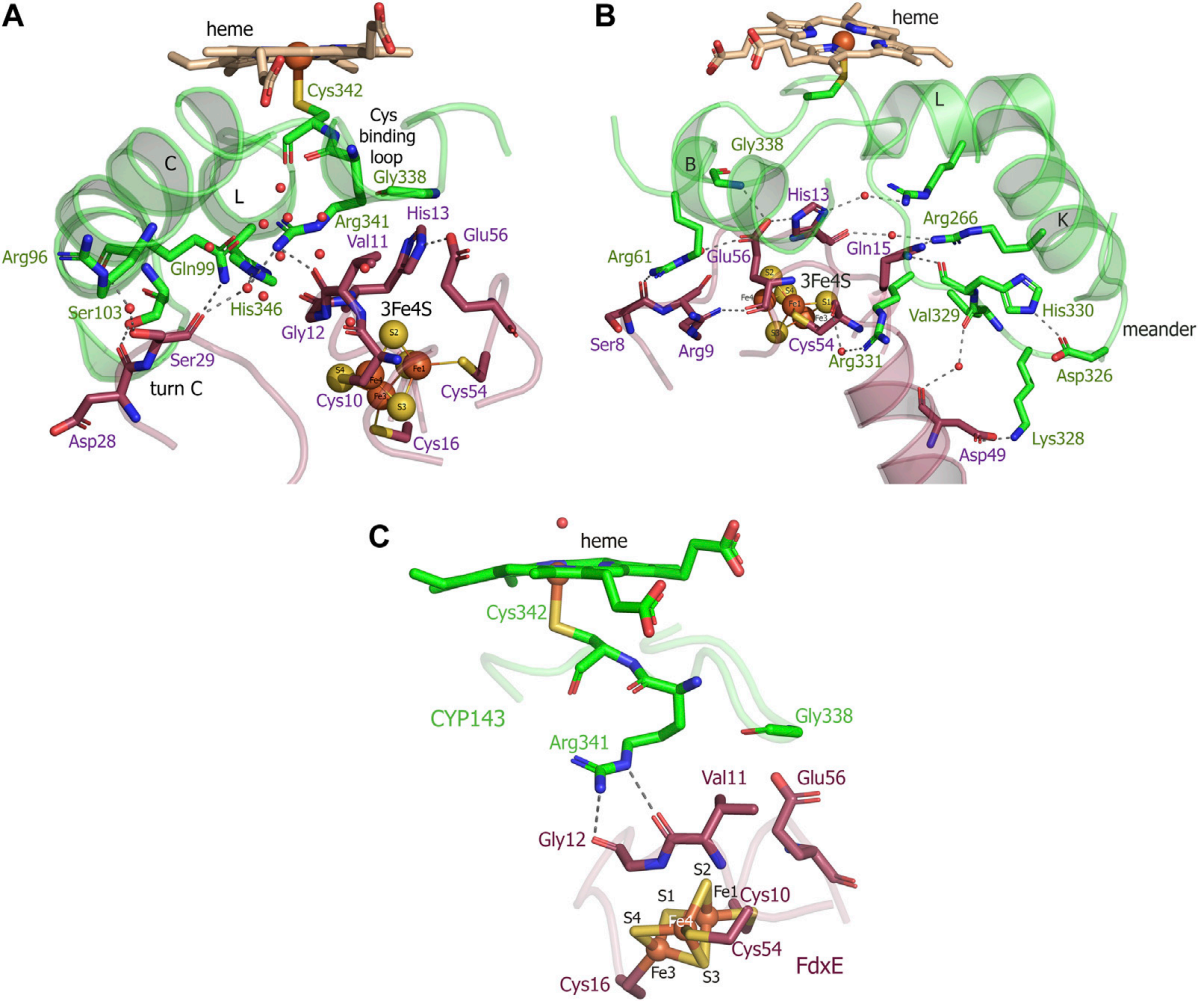
**(A,B)** Views of the interactions between FdxE and CYP143. **(C)** Residues important for mediating electron transfer coupling between the donor and acceptor. Coupled atoms were computed separately by selecting either Fe1 or Fe4 atoms of the [3Fe-4S] cluster as the donor using HARLEM. FdxE is colored raspberry and CYP143—green.

The electron density of the iron–sulfur cluster of FdxE clearly identifies a [3Fe–4S] cluster coordinated by Fe—S bonds to cysteines Cys10, Cys16, and Cys54. The [3Fe–4S] cluster has typical cuboidal geometry with similar ∼2.3 Å bond lengths between the cysteinyl sulfur and Fe (Supplementary Figure S1). A residue His13 of the CXX**H**XXC(X)nCP motif is not ligated to the [3Fe–4S] cluster (Figure 4). Instead, the N1 atom of the imidazole ring of His13 forms a hydrogen bond with the O∑1 atom of Glu56 from the opposite loop around the cluster. The other interactions of His13 are water-mediated contacts with the CYP143 residues: the N3 atom of the imidazole ring—with Arg349 of the L helix, and the main chain oxygen—with Arg266 from the K helix.

The side chain of Glu56 of FdxE is in direct contact with Gly338 of the Cys-coordinating loop of CYP and involved in a water-mediated interaction with the side chain of Arg61 from the B helix of CYP143. The main chain of Glu56 is stabilized by the internal interaction with Arg9 that precede cluster-coordinating cysteine. The CYP residue Arg61 also forms a weak hydrogen bond (a distance of 3.1 Å) with the main chain oxygen of Ser8 in turn A of FdxE. In a complex, His13 and Glu56 residues are positioned right below the Cys-coordinating loop of CYP.

In addition, the complex is stabilized by the hydrogen bond between Arg266 of the CYP143 K helix and Gln15 preceding the cluster coordinating Cys16 of FdxE (Figure 4B). Although the side chain of Gln15 modeled in two conformations, it does interact with CYP, either with Arg266 or with the main chain of His330 from the meander.

A meander region of CYP is sandwiched between a K helix of CYP and helix 2 of FdxE. The inner salt bridge in the meander, between residues Asp326 and His330, maintains its specific conformation so that Lys328 forms a salt bridge with Asp49 of FdxE (Figure 4B). Additionally, this interaction region is stabilized by two water-mediated contacts: between the main chain oxygen of Asp49 of FdxE and the main chain oxygen of Val329 of CYP, and between the main chain oxygen of Cys54 coordinating the cluster of FdxE and side chain of Arg331 of CYP.

The loop coordinating heme of CYP is adjacent to the meander region and involved in the interaction with the redox partner. Here, in addition to aforementioned Gly338, the residue Arg341 immediately preceding the proximal ligand of heme is H-bonded to Val11 of FdxE.

Finally, the interaction spot between redox partners is also observed on the periphery of the interaction interface (Figure 4A). The turn C of FdxE perfectly fits between two adjacent helices C and D of CYP, forming a hydrogen bond between Asp28 of FdxE and Ser103 of CYP. The intermolecular interaction is additionally stabilized by a water-mediated interaction of Ser29 of FdxE and Arg96 of CYP. The same Ser29 also forms a weak H-bond (distance is 3.1Å) with Gln99 of CYP and water-mediated contacts with Arg341 and His346 of CYP. Notably, the water chain observed in this region fills the interaction interface between the loop harboring the Cys10 ligand of the [3Fe–4S] cluster and the C helix of CYP. Overall, the hydrogen bonding interactions predominate in the formation of the FdxE–CYP143 complex consistent with the obtained thermodynamic data.

In the absence of the experimental information of which Fe of the [3Fe−4S] cluster is favored for ET, there are two equivalent donors—Fe1 and Fe4 —having similar distance (∼16.8Å) to the heme iron. The predicted best pathways were computed using the HARLEM (HAmiltonians for Research of LargE Molecules) program (http://www.harlemprog.org). Electrons could flow from Fe1-S2 to the Gly12—Val11 peptide or, alternatively, from Fe4 to the Cys10 –Val11 peptide and then *via* Arg341 and cysteinyl ligand (Cys342) directly to the heme iron. The residues having atoms important for mediating ET coupling between the donor and acceptor are shown in Figure 4C. Both pathways have a similar ET log10 rate of 4.33 s^−1^ and 4.77 s^−1^ for Fe1 and Fe4 donor atoms, respectively, computed using the Dutton model in HARLEM. Based on these calculations either Fe is suitable for ET, and further studies are required to determine the contribution of each Fe of the cluster. Taken together, the precise positioning of the cluster right below the heme-binding loop, the short, efficient, and coupled pathway for electron flow to the heme iron, indicates that the complex between two proteins is specific.

### CYP143 structural changes for complex formation

**FIGURE 5:**
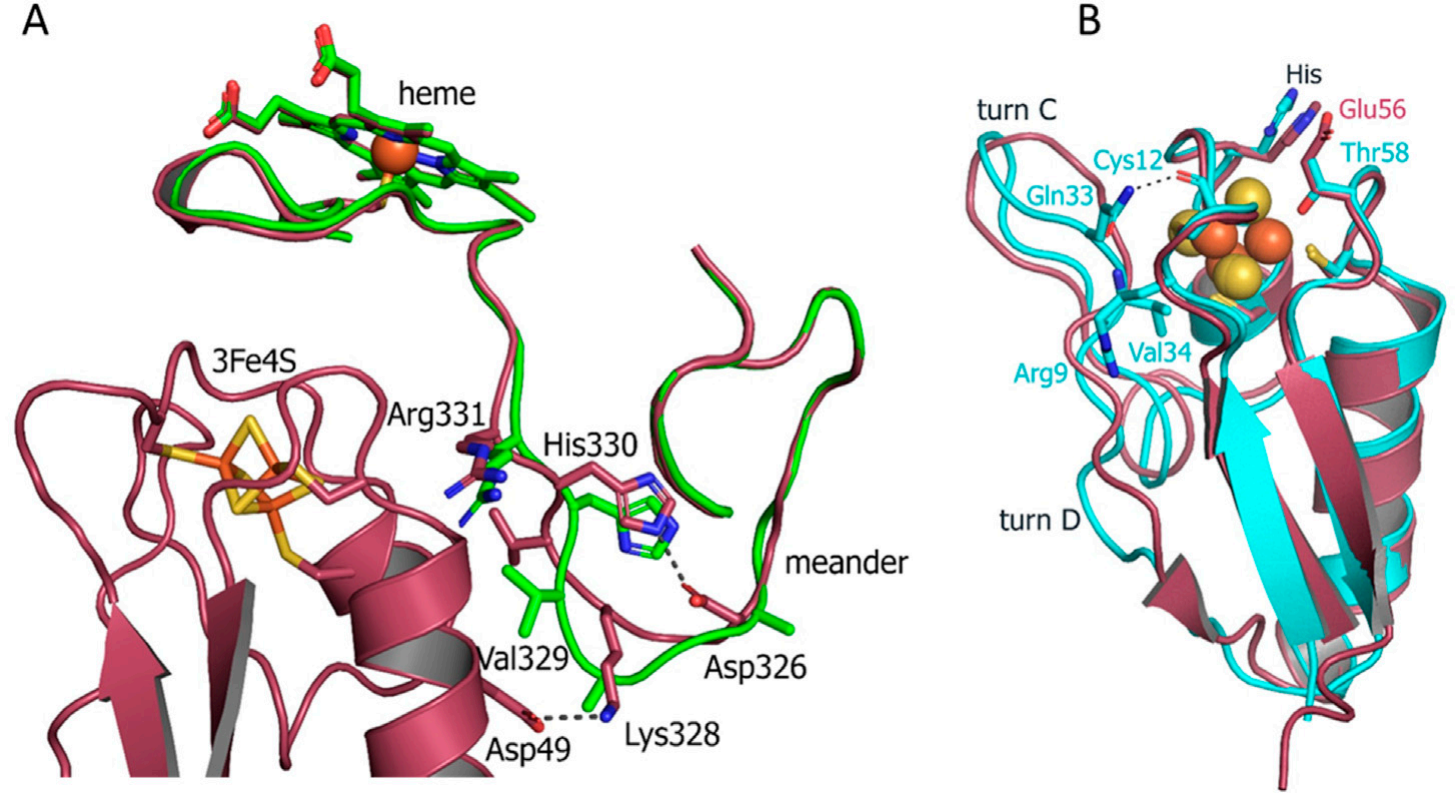
**(A)** Superposition of the CYP143 structure alone (green ribbon) with the structure of the FdxE−CYP143 complex (raspberry) detailing the meander region. **(B)** Superposition of Fdx (cyan) and FdxE (raspberry).

To visualize structural changes in CYP143 induced by the interaction with FdxE we solved the CYP143 structure alone at similar resolution (1.4 Å). It is worth mentioning that attempts to crystallize FdxE alone were unsuccessful. CYP143 structure is conforming to the typical cytochrome P450 fold with 12 α-helices (A–L) and 4 β-sheets. In the absence of the ligand, protein crystallized in open conformation with the heme cofactor accessible to the bulk solvent. The iron of the heme is hexacoordinated by the invariant Cys342 and a water molecule. This water is stabilized by the H-bond with glycerol molecule from a cryoprotectant solution. Superposition of the CYP143 structure with the CYP143 part of the complex structure shows RMSD = .932Å for all atoms and reveals a different position of the lower part of the meander (Figure 5A). In the CYP143 structure alone, the meander is away from the K helix, while in the complex it moves closer and sandwiched between helix 2 of FdxE and K helix of CYP. The relocation distance of 5.5 Å is observed for the Gly327 Ca atom. Upon complex formation the salt bridge is formed between Asp326 and His330 shaping the meander for the interaction with ferredoxin by a salt bridge between Lys238 of CYP and Asp49 of FdxE (Figure 5A) as well as water-mediated contacts described above.

A closer examination of the interacting regions of CYP143 reveals a different side chain conformation of the charged residues involved in intermolecular contacts with Fdx in a complex structure Gln99 of the C helix, and Arg266 of the K helix.

With the CYP143 structure alone in hands, we calculated its electrostatic surface potential (Supplementary Figure S5A). The local proximal area involved in the interaction with FdxE is positively charged, despite overall theoretical pI = 6.8. With FdxE being negatively charged (Supplementary Figure S5B), one could suggest that electrostatic patches enhance the presence of encounter states.

### Small-angle X-ray scattering analysis (SAXS)

**FIGURE 6:**
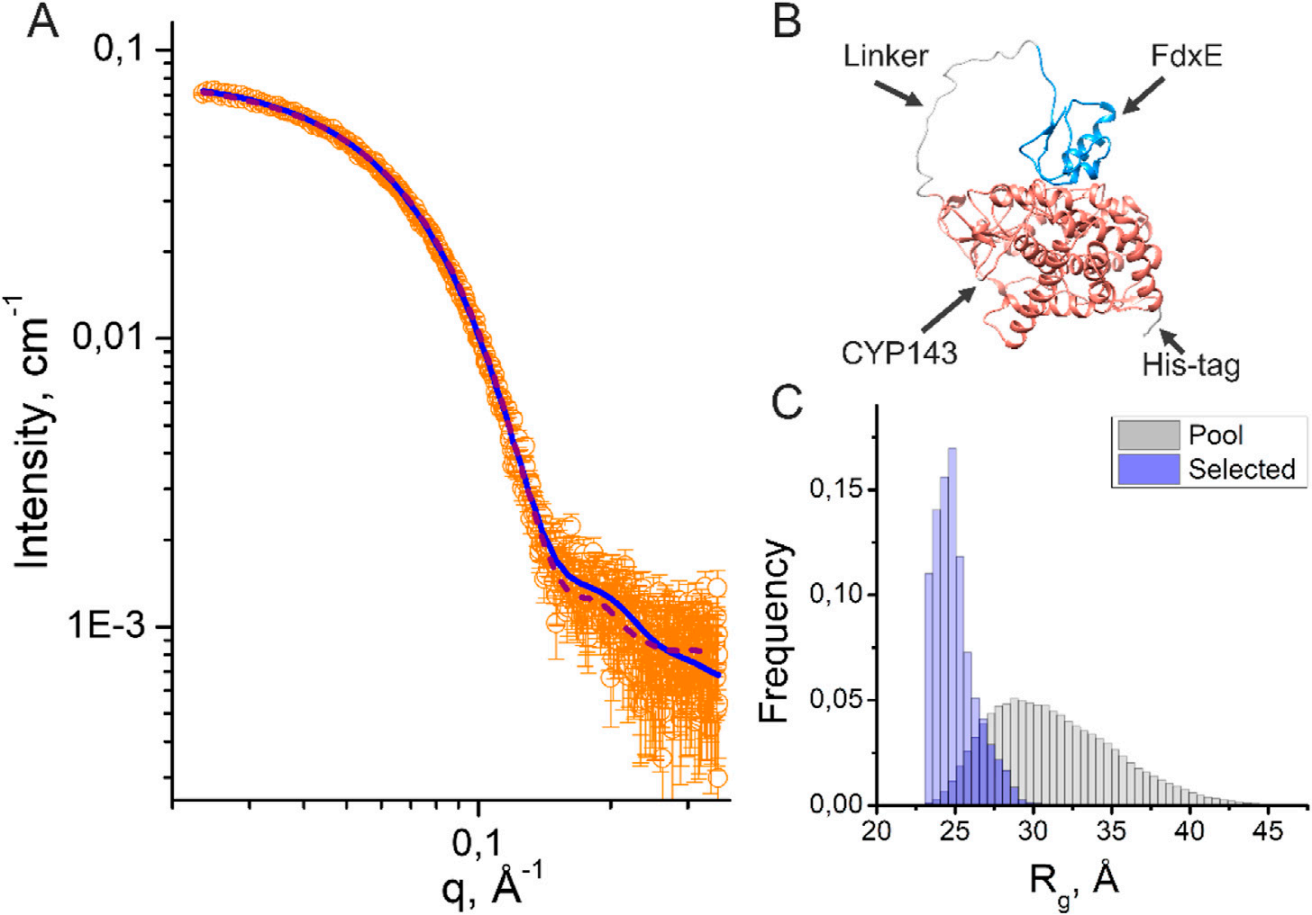
Results of SAXS data analysis of the fusion FdxE−CYP143 complex. **(A)** SAXS I(q) profile (orange circles) and their approximations corresponding to the PDB models obtained by using CORAL (purple dashed line) and EOM (blue solid line); **(B)** CORAL model of the fusion FdxE-CYP143 complex; **(C)** Rg distributions for the initial pool (gray histogram) and for a set of selected conformations (blue histogram) in the EOM (Supplementary Figure S6).

To obtain information about the structure and conformational flexibility in solution, the fusion complex FdxE−CYP143 was investigated by SAXS. Assuming that CYP143 and FdxE positions are fixed at their crystal positions, we performed SAXS-based modeling using the CORAL program (Petoukhov et al., 2012). The obtained structure (Figure 6B) approximates SAXS data well (χ^2^ = 1.41, Figure 6A and Supplementary Table S2). It strongly indicates that the FdxE−CYP143 complex in solution is similar to that in the crystal structure, and, consequently, the contacts between CYP143 and FdxE are not an artifact of the crystallization.

Additionally, we used the ensemble optimization method (EOM) (Tria et al., 2015) to fit SAXS experimental data with an ensemble of proteins with variable positions of CYP143 and FdxE. The EOM is suitable for describing the distribution of the relative position of two domains connected by a linker, even if these domains differ significantly in molecular weight (Khramtsov et al., 2020). The EOM generated a large pool of possible conformations (that has a distribution of *R*_*g*_ shown in Figure 6C) and then it selected a subset of conformations that minimizes the discrepancy χ^2^ between theoretical and experimental SAXS data. The distribution of *Rg* for the selected subset of conformations corresponds to the smallest sizes compared to the initial pool of conformations (Figure 6C), indicating that the majority of the protein molecules in solution are in the most possible compact conformations.

The multistate model with equal weights generated by the EOM (Supplementary Figure S6A, χ^2^ = 1.16) contains four out of five structures similar to the position of FdxE in the crystal structure. Together with Rg distribution, the multistate model indicates that the majority of the protein molecules form complexes similar to those in the crystal structure.

A separate EOM run with variable weights (χ^2^ = 1.14) produced three models with 67%, 22%, and 11% occupancies (Supplementary Figure S6B). All models are located in close proximity to the position of Fdx in the stereospecific complex. Notably, the closer position corresponds to the higher occupancy of the model. Possibly, the variations in models may account for encounter states presented in the ensemble (Hiruma et al., 2013) (Andralojc et al., 2017).

### Comparison of Fdx and FdxE structures

To understand the specificity of two ferredoxins from Mtb, we compared their structures. Although both ferredoxins have the same fold and share the same cluster coordinating motif, some differences are clearly seen. The structures were aligned with Cα RMSD = 1.614 Å. Two main differences are the absence of turn D in Fdx and the position of turn C (Figure 5B). The latter is of particular importance as it is involved in the interaction with a redox partner. As mentioned previously, not only the conformation but the amino acid composition of the turn C also differ between two ferredoxins. In the Fdx structure, turn C is additionally stabilized by the interactions with turn A residues: backbone Val34 with Arg9 and Gln33 with backbone Cys12.

The His13 residue from the motif interacts with the Glu56 residue in the FdxE structure, while the corresponding Thr58 residue in Fdx is pointed toward the cluster with the distance to the S2 = 3.7 Å.

The residues interacting with the redox partner are also different between two ferredoxins. Specifically, Val11 implicated both in the interaction with the redox partner and electron flow corresponds to Gln13 in Fdx. The surface residues Ser8, Arg9 from turn A, and Gln15 from helix 2 of FdxE correspond to Asp10, Leu11, and Met17 in Fdx, respectively (Figure 2). So the polar contacts in these regions are opposite in charge for two ferredoxins. In addition, Asp49 in FdxE forms a salt bridge with Lys328 of the adjusted meander of CYP replaced by His51 in Fdx, further revealing their specialization.

## Discussion

In this work, we focused on two 3Fe–4S ferredoxins from Mtb associated with CYPs. We solved crystal structures for both of them, one for ferredoxin alone and a second in the complex with CYP143 (Supplementary Table S2). For a better understanding of the redox partner-induced structural changes, we solved a crystal structure of CYP143 alone. With this set of structures, we noticed the rigid structure of small ferredoxins, but adaptations for the interaction are seen in CYP.

We explored the genetic context for both ferredoxins. Evolutionary advantages may be gained by colocalizing redox partner genes into the same operon and/or adjacent to each other. A 3Fe−4S ferredoxins of *Mycobacterium marinum* (Child et al., 2018), *Streptomyces griseolus* (O'Keefe et al., 1991), and *Rhodopseudomonas palustris* (Bell et al., 2006; Zhang et al., 2014) are associated with cytochrome P450 enzymes with demonstrated *in vitro* catalytic efficiency.

In Mtb, only two ferredoxin genes are organized in this manner, albeit with a notable difference: the reading frame for *Rv1786* is in the opposite direction relative to *CYP143* (Supplementary Figure S3A), while it is the same for *Rv0763c* and *CYP51* (Supplementary Figure S3B). Of note, other species homologs of *Rv1786* are organized in one direction within the same operon with *CYP*, indicating functional relevance (Supplementary Figure S3A).

In general, CYP51−Fdx fusions are not rare in nature and can be found in different bacterial phyla (Jackson et al., 2002; Lamb et al., 2021) including Actinobacteria to which Mtb belongs*.* Native fusion McCYP51−Fdx from *Methylococcus capsulatus* has 3Fe–4S type of ferredoxin and was hypothesized to emerge due to the mutation of a CYP51 non-sense codon leading to translational read-through to an adjacent ferredoxin (Jackson et al., 2002). The interactions between redox partners within McCYP51−Fdx were described as transient with various orientations of the ferredoxin molecule (Hargrove et al., 2022). Of note, CYP51−Fdx fusions still require a reductase component for catalysis. In case of *Rv0763c* homologs (Supplementary Figure S3), the fusion with reductase was observed in *Myxococcus xanthus DK 1622* further expanding the fusion capacity of ferredoxins.

We were unable to find CYP143-related fusions with *Rv1786*. However, the search using the CXXHXXC(X)nCP motif revealed a native fusion in mercury methylating marine bacteria (Lin et al., 2021) which is distantly related to *Rv1786* (30.85% homology), where the N-term ferredoxin domain is fused to CYP. Overall, the fusion strategy is common in bacteria, including pathogenic species. Some relevant examples of fusions are discussed as follows.

Phthalate family oxygenase reductase (PFOR)-like fusion enzymes comprising the CYP116 family are self-sufficient, e.g., NAD(P)H binds directly to the reductase domain and electrons are transferred through FMN, then the 2Fe–2S center and onto the P450 heme iron. CYP116 PFOR fusion proteins are found in *Rhodococcus, Burkholderia, Ralstonia, Labrenzia, Acinetobacter, Toriphiles,* and other species. Recently, the 3D-structure of CYP116B46 from *Tepidiphilus thermophilus* was solved (Zhang et al., 2020), revealing the overall arrangement of the redox chain. In the structure, the 2Fe–2S ferredoxin domain is oriented toward the reductase domain with the distance between FMN and 2Fe–2S cofactors being 7.9 Å, which represents the initial arrangement for the electron transfer. The ferredoxin domain must have sufficient mobility upon reduction for the next step where it interacts with the CYP domain. The molecular dynamic simulation studies confirm the transit of ferredoxin from “distal” to “proximal” conformation enabling efficient electron transfer from the reduced ferredoxin to the heme domain (Wang et al., 2021). However, there is still the possibility of a dimeric complex [as for P450BM3 (Neeli et al., 2005)] with the inter-monomer electron transfer. Of note, the complex between the ferredoxin domain and CYP domain in CYP116B46 was modeled using the reference structure of the designed fusion of native redox partners, CYP11A1 and adrenodoxin, we solved earlier (Strushkevich et al., 2011). Up to date despite the ferredoxins biodiversity in CYP-mediated reactions, the crystal structures of complexes with CYPs are limited to 2Fe–2S ferredoxins.

In this work, we designed a fusion FdxE−CYP143 of Mtb using a natural linker from the PFOR of *Rhodococcus* sp. *NCIMB 9784* which we used for the fusion of mammalian redox partners (Strushkevich et al., 2011). Protein crystallography allowed us to obtain the atomic structure of the fused FdxE−CYP143 complex and SAXS analysis further confirmed the formation of the complex in solution. The matter remains to what extent the artificial fusion represents the structure of the native FdxE−CYP143 complex. Previously described complexes of CYPs with ferredoxins [see for instance (Strushkevich et al., 2011; Hiruma et al., 2013)] closely resemble the studied FdxE−CYP143 complex with proximal location of ferredoxins. The linker between FdxE and CYP143 is sufficiently long to allow mutual orientation of protein partners. It is also not resolved in electron densities that indicate its flexibility. These facts demonstrate that the linker does not impose limitations on protein complex formation. Therefore, we argue that the obtained structure of the fused FdxE−CYP143 complex represents a native complex of the two proteins.

**FIGURE 7:**
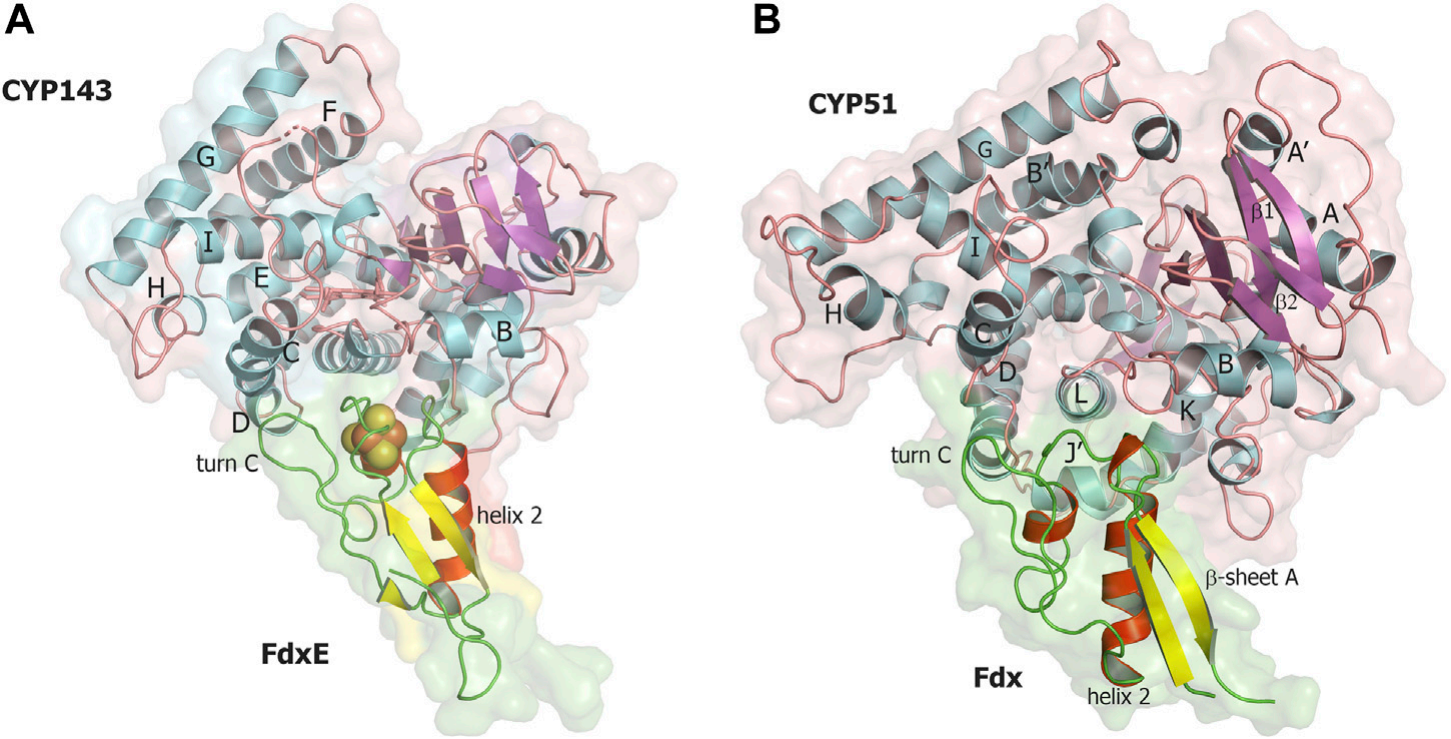
Comparison of two complexes of CYP with 3Fe–4S ferredoxins. **(A)** FdxE−CYP143 complex obtained in this work, **(B)** CYP51 – Fdx modeled with AlphaFold2. CYP143, and CYP51 are colored by secondary structures in cyan, magenta, and salmon for the helix, sheet, and loop, respectively; FdxE and Fdx are colored in red, yellow, and green for helix, sheet, and loops, respectively.

Using this fusion strategy, we were able to characterize the FdxE−CYP143 complex and highlight certain features related to the 3Fe–4S type of ferredoxin. However, how the specificity of ferredoxins in CYP-mediated reactions is realized and controlled in different bacteria is still an open question. We modeled the CYP51–Fdx complex using AlphaFold2 (Mirdita et al., 2022) and compared it with our experimentally obtained structure of the FdxE−CYP143 complex (Figure 7).

In the modeled CYP51–Fdx complex, the position of Fdx is shifted to the C helix so that the Cys-coordinating loop is not right above the cluster but shifted and above the turn C of Fdx (Figure 7B). The CYP51 proximal surface is strikingly different from CYP143 as the MtbCYP51 structure has quite unique features: a bent I helix and an open conformation of the BC loop (Podust et al., 2001) and, as a consequence, the repositioning of the C- and H-helices and the adjacent loops. The observed docking position of Fdx ferredoxin could also be related to its unusual turn C conformation, affecting the interaction with CYP. In addition, CYP51 has a J′ helix missing in the CYP143 structure. These structural differences along with amino acid sequence differences between Fdx and FdxE (Figure 2) led to deviations in the interacting residues between redox partners. One can speculate that conformational changes would take place on the proximal surface of CYP51 upon Fdx binding and the CYP51 fold would be more similar to other CYP51 homologs. A recently reported CYP51 structure from *Mycobacterium marinum* (Mohamed et al., 2022) is similar to MtbCYP51, suggesting specificity of bacterial cognate ferredoxins.

In cytochrome c reductase activity assay, Fdx and FdxE demonstrated different preferences for the reductases (Ortega Ugalde et al., 2018). Furthermore, FdxE is significantly less sufficient in supporting *in vitro* catalytic activities of Mtb CYP124A1 (Ortega Ugalde et al., 2018) and our own unpublished data), CYP125A1, and CYP142A1 and does not support CYP121 catalysis (Ortega Ugalde et al., 2018), suggesting that it is less promiscuous and more specific than Fdx. A summary of the *Rv0763c* and *Rv1786* gene expression using MicrobesOnline (Dehal et al., 2010) also reveals that they are induced and downregulated at different stress conditions (Supplementary Figure S8). From this analysis, it appears that *Rv0763c* and *Rv1786* cannot substitute each other in supporting respective CYP-mediated reactions, which is feasible considering their organization in the *M. tuberculosis* genome. Further studies should be focused on the search for potential substrates for CYP143 to obtain more information about the regulation of interaction between these redox partners.

In summary, many ferredoxin genes associated with the CYPome in bacteria provide more specific control over synthesized metabolites under different conditions. However, these systems have yet to be characterized in detail. We believe that our data shed light on the structural preferences driving protein–protein interactions within these electron transfer complexes.

## Methods

### Cloning, expression, and purification of Fdx, FdxE, CYP143, and FdxE‐CYP143 fusion

We amplified the Fdx coding sequence with a 6-His tag on the C-terminus using the atc​ata​tgg​gct​atc​gag​tcg​aag​c forward primer and tag​gat​cct​taa​tgg​tga​tgg​tga​tgg​tgc​tct​ccc​gtt​tct​cgg​atg reverse primer. *Mycobacterium bovis* BCG genomic DNA was used as a template instead of *M. tuberculosis*, since the Fdx sequences are identical in both genomes. The PCR product then was cloned into a pET11a vector using NdeI and BamHI restriction enzymes. The insert sequence was validated by sequencing. The FdxE was cloned similarly into the expression vector pET11a.

The Fdx was expressed in *E. coli* BL21 (DE3). The cells, harboring the expression vector, were grown in TB medium at 37°C with an addition of 100 ug/mL ampicillin and .5 mM FeCl_3_. When the culture reached OD_600_ ∼.7, Fdx expression was induced with .7 mM IPTG. The cells were harvested after incubation at 22°C for 16 h post-induction. The cells were re-suspended in buffer A (50 mM TrisHCl, pH 7.5, 300 mM NaCl, 20% w/v glycerol) containing 1 mM PMSF and disrupted with Emulsiflex C3 homogenizer. The lysate after centrifugation was applied on an Ni-NTA agarose column, followed by washing with buffer A containing 60 mM imidazole, and gradient elution with buffer A containing 500 mM imidazole. Fractions with an absorption peak at 412 nm were diluted 30-fold with buffer B (10 mM TrisHCl, pH 7.5, 20% w/v glycerol) and applied on a DEAE-Sepharose column. After a 20 CV washing step with buffer B, the protein was eluted with a linear gradient of buffer C (50 mM TrisHCl, 1 M NaCl, 20% w/v glycerol). Fdx concentration was calculated using molar extinction 11,300 M^−1^cm^−1^ at 412 nm (McLean et al., 2006).

FdxE was expressed in *E. coli* JM109. Overnight culture (3 mL) was used to inoculate .5 L of TB-medium containing 100 mM potassium–phosphate buffer, pH 7.4, and ampicillin (100 μg/mL). The mixture was incubated in a thermostatic orbital shaker at 37°C and 180 rpm. After reaching OD_600_ ∼ .4, FdxE expression was induced by adding IPTG (.5 mM) and ampicillin (100 μg/mL). A solution of FeCl_3_ (100 μg/mL) was also added at this point. After 24 h of incubation at 26°C and 100 rpm, the cells were collected by centrifugation (8000 g, 10 min). The pellet was re-suspended in a 50-mM potassium–phosphate buffer, pH 7.4, containing 20% glycerol, .1 mM EDTA, and .5 mM PMSF. Cell suspension was sonicated in an ice-water bath (7x1-min pulses with 1-min intervals). The suspension was centrifuged for 1 h at 20,500 rpm and the supernatant was applied to a column with DEAE-Sepharose equilibrated with buffer A (50 mM potassium–phosphate buffer, pH 7.4, containing .1 mM EDTA). The column was washed with 2–3 volumes of buffer A, and then with 10 volumes of buffer A containing 15 mM NaCl. FdxE was eluted from the column with buffer A, containing 200 mM NaCl. Eluted fractions were applied to a Superdex 200 16/60 column equilibrated with buffer A, containing 200 mM NaCl. The colored fractions containing FdxE were collected.

The constructs for CYP143, CYP143-Avi-tag at the C-terminus, and FdxE−CYP143 fusion were cloned in the pCW-LIC vector by LIC and co-expressed with GroEL-GroES in an *E. coli* strain DH5a in Terrific broth medium at 26°C with shaking at 100 rpm for 48 h after induction. In the case of CYP143-Avitag, TB medium was supplemented with biotin (50 µM). Induction was performed at OD_600_ ∼ .8 by IPTG (.5 mM) and arabinose (2 g/L). .65 mM δ-aminolevulinic acid was added to the growth medium as a heme precursor. For FdxE−CYP143 fusion, FeCl_3_ (20 µM) was added. Cells were collected by centrifugation (3,500 rpm for 20 min at 4°C) and re-suspended in 50 mM Tris-HCl pH 7.4 buffer, containing .3 M NaCl and .5 mM PMSF.

FdxE−CYP143 fusion was purified by metal affinity and ion-exchange chromatography. Cells were lysed by passing through an Emulsiflex C5 Homogenizer (Avanti, Canada) twice and then centrifuged to remove membrane fraction (22,000 rpm, 4°C for 1 h). The supernatant was loaded on an IMAC column (5 mL Ni-NTA His-Trap HP, Cytiva). The column was washed with 15 CV of buffer 50 mM Tris-HCl, pH 7.4, .3 M NaCl, 25 mM imidazole, and the protein was eluted with a linear gradient of imidazole (.025–.5 M). Red fractions were pooled and purified on a Q-Sepharose column (Source 30Q, Cytiva) pre-equilibrated with buffer 10 mM Tris-HCl, pH 7.4. After washing with 15 CV of buffer containing .1 M NaCl, the protein was eluted with a linear gradient of NaCl (.10—1 M). The protein fractions were analyzed by SDS-PAGE and spectrophotometrically, then pooled and concentrated; glycerol was added for storage. CYP143 was purified similarly. Biotinylated CYP143 was purified by affinity chromatography followed by dialysis. Proteins were frozen in liquid nitrogen and stored at −80°C.

The absolute spectra of Fdx, FdxE, CYP143, and FdxE−CYP143 fusion proteins were recoded to ensure the proper folding of the studied proteins. The CO spectrum was recorded according to (Omura and Sato, 1964) for CYP143 and FdxE−CYP143 prior to the experiments to confirm that the proteins are in their functional P450 form (Supplementary Figure S9).

The concentration of Fe in the studied proteins was determined using an inductively coupled plasma mass spectrometer Agilent 7500 ICP-MS (Agilent Technologies, Santa Clara, CA, United States). Protein solution of 100 μL was microwave-digested with .5 mL of high-purity concentrated HNO_3_ (Merck, Germany) and .1 mL of high-purity concentrated H_2_O_2_ (Fisher Chemicals, United Kingdom) in a microwave digestion system MARS (CEM, United States) at 1,600 W for 4 min. Once cooled, digests were made up to 10 mL using deionized water, and the solutions were subjected to element determination by ICP-MS. Commercially available periodic table mix 1 for ICP (Sigma-Aldrich, No. 92091) was used for standardization of the calibration curve. ICP-MS acquisition parameters: Babington nebulizer; spray chamber Quartz cooled to 2°C; interface cones Ni; cell gas He; integration time .3 s; number of points per mass 3; and repetition 3. The predicted amount of Fe in the CYP143 sample is 8.9 µg, the experimental value is 9.28 ± 3.28 indicating 1.04 ± .37 iron within the heme group as expected. For the FdxE−CYP143 fusion, the predicted amount of Fe in the sample is 35.67 μg, while the experimental value is 33.60 ± 4.65 µg. The measured amount of Fe is 3.77 ± .52, indicating four Fe atoms per molecule of the fusion protein that corresponds to one Fe atom within the heme and three Fe atoms in the [3Fe–4S] cluster of FdxE.

### Crystallization of Fdx, CYP143, and FdxE−CYP143

For Fdx crystallization, we used a sitting drop method. Crystals grew at 20°C in drops containing 1 ul of the protein at concentration 9 mg/mL and 1 ul of .1 M Tris-HCl pH 8.5, .2 M MgCl_2_, and 25% w/v PEG3350.

CYP143 (150 µM) and FdxE−CYP143 (200–250 µM) were crystallized in a 96-well plate using a sitting-drop method with commercially available kits from Qiagen (NeXtal Classics II screen) and Molecular Dimensions (structure screens 1 and 2) at 20°C with a 1:1 protein/mother liquor ratio with a ligand concentration of 1–2 mM. Red crystals of CYP143 appeared overnight and FdxE−CYP143 in 2 weeks. The best crystals of CYP143 and FdxE−CYP143 grew in .2 M sodium chloride, .1 M Bis-Tris pH 6.0, 25% (*w/v*) PEG3350.

### Surface plasmon resonance

SPR analyses were carried out using the optical biosensors BiacoreX100, Biacore 3,000, and Biacore 8K (Cytiva, Marlborough, MA, United States) and sensor chips of the SA series S type (Cytiva, Marlborough, MA, United States) at 25°C. The buffer PBS (10 mM Na_2_HPO_4_, 1.8 mM KH_2_PO_4_, 137 mM NaCl, and 2.7 mM KCl, pH 7.4) (Cytiva) was used as a running buffer for CYP143 immobilization and SPR analysis. Before immobilization, the chip surface was conditioned with 1M NaCl and 50 mM NaOH solution injection at a flow rate of 30 ul/min for 1 min. Next, 480 nM solution of biotinylated CYP143 with AVI-tag in PBS buffer was injected into the working channel of the biosensor for 7 min at a flow rate of 10 μL/min. The mean final level of immobilization was 5,500 ± 500 RU. A reference channel without immobilized CYP143 was used to correct the effects of the non-specific binding of analytes to the chip surface.

### Estimation of kinetic and equilibrium parameters

To assess the parameters of protein–protein interactions, FdxE was injected at various concentrations (5–75 nM) through the cell with CYP143 immobilized on the SA chip. The resulting sensorgram represents the difference between the experiment (with immobilized CYP143) and the control (without CYP143) channels. The flow rate was 30 μL/min and the contact time was 2 min. No regeneration of the chip surface was required as the complex dissociates completely within 2 min. The resulting sensorgrams were processed in the BIAevaluation 4.1.1 software using the 1:1 interaction model with mass transfer.

The τ_1/2_ values characterizing half-time dissociation of a protein–protein complex were calculated from the k_off_ values according to the following equation:

$$\tau_{1/2}=\frac{\ln2}{k_{off}}.$$ (1)

### Thermodynamic parameters of CYP143 and FdxE interaction

Sensorgrams were obtained at temperatures of 10, 15, 20, 25, 30, and 35°C. FdxE concentrations, the flow rate, and contact time were the same as described previously.

The Gibbs free energy (ΔG) was calculated from Eq. 2 using the Kd value obtained.

ΔG=RT⁡ln⁡Kd, (2)

where T is the absolute temperature (°K), R — the universal gas constant (J*×mol^−1^*×K^−1^), the Kd — the equilibrium dissociation constant of the protein–protein complex M).

Enthalpy change (ΔH) was determined by plotting lnK_d_ *versus* 1,000/T (Van’t Hoff plot) according to the linear form of the Van’t Hoff Eq. 3, using the slope of the linear regression line (ΔН/R).

$$\ln Kd=\left(\frac{\Delta H}{R}\right)\left(\frac{1000}{T}\right)-\left(\frac{\Delta S}{R}\right).$$ (3)

The entropy change (−TΔS) was calculated from the following equation:

ΔG=ΔH−TΔS. (4)

### Data collection and X-ray structure determination

Diffraction data were collected at the European Synchrotron Radiation Facility (ESRF) beamlines ID23-1, ID30A1, and ID30B for CYP143, Fdx, and FdxE−CYP143, respectively. The data collection strategy was optimized in BEST (Popov and Bourenkov, 2003; Bourenkov and Popov, 2006). To increase data completeness for the CYP143 P1 crystals, two datasets from the same crystal were collected using different kappa angles.

All data were processed in the XDS software package (Kabsch, 2010). Processed data were corrected for anisotropy using the STARANISO server (Vonrhein et al., 2018) (http://staraniso.globalphasing.org/cgi-bin/staraniso.cgi).

A local mean I/σ(I) value of .50 was used to determine the anisotropic diffraction-limit surface.

The phase problem for CYP143 was solved in the automatic molecular replacement pipeline MoRDa (Vagin and Lebedev, 2015), where the structure of CYP101D2_Y96A_ (PDB ID: 4DXY (Bell et al., 2013)) was used as a starting model. The obtained space group was P1 with one molecule per asymmetric unit. Initially, molecular replacement gave the model with poor-quality maps and the solution was further optimized in the Morph model from Phenix (Terwilliger et al., 2012; Liebschner et al., 2019). The resultant model was than rebuilt using the ARP/wARP web service (Tilley et al., 2001; Chojnowski et al., 2020).

The phase problem for Fdx was solved in the automatic molecular replacement pipeline MoRDa (Vagin and Lebedev, 2015) from the CCP4 Online web service (Krissinel et al., 2018), where the structure of [4Fe-4S] ferredoxin [PDB ID: 1VJW (Macedo-Ribeiro et al., 1996)] was used as a starting model. The obtained space group was H32 with one molecule per asymmetric unit. Initially, molecular replacement gave the model with poor-quality maps and the solution was further optimized in the Morph model from Phenix (Terwilliger et al., 2012; Liebschner et al., 2019). The resultant model was then rebuilt in phenix AutoBuild (Chojnowski et al., 2020).

To solve the phase problem for the FdxE−CYP143 complex, polyala models of the refined CYP143 and Fdx were used for molecular replacement in Phaser (McCoy et al., 2007). The space group for the complex was P1 and contained one molecule of both CYP143 and Fdx in ASU. The resultant model was then rebuilt in phenix AutoBuild (Terwilliger et al., 2008).

For all the models, multiple rounds of TLS-refinement (Painter and Merritt, 2006) were conducted in phenix.refine (Afonine et al., 2012) and refmac5 (Murshudov et al., 2011). Interactive refinement was performed in Coot (Emsley et al., 2010). The quality of the final models was analyzed using phenix. molprobity (Williams et al., 2018). The Fe ion in the Fdx structure was validated using Check My Metal web service (Handing et al., 2018) and anomalous difference map, built with FFT from CCP4 (Winn et al., 2011). Data collection and final refinement statistics are given in Supplementary Table S1. Figures were rendered in PyMOL. 2Fo-Fc and anomalous difference Fourier maps on cofactors are shown in Supplementary Figure S10.

Structures of FdxE−CYP143, CYP143, and Fdx have been deposited in the Protein Data Bank (PDB) under the accession codes 8AMQ, 8AMO, and 8AMP, respectively.

### Small-angle X-ray scattering measurements and data processing

SAXS measurements were carried on the BM29 BioSAXS beamline (ESRF, Grenoble, France) (Pernot et al., 2013). All measurements were performed with 100% of beam intensity at a wavelength of .9918 Å (12.5 keV). Initial data processing was performed automatically using the EDNA pipeline (Incardona et al., 2009; Brennich et al., 2016). SAXS profiles were obtained at a protein concentration of 2.2 mg/mL, which was small enough to neglect the influence of the structure factor S(q) on the scattering curves (Poster Sessions, 2014; Zabelskii et al., 2018), in contrast to systems with relatively high sample concentrations [∼1% or more (Murugova et al., 2022). Exposure time was 8 s for the sample and 205 s for the buffer.

SAXS profile *I*(*q*) was processed using ATSAS (Franke et al., 2017) software suite. The SAXS curve obtained for the fused complex FdxE−CYP143 has a wide Guinier region (Supplementary Figure S7A) that confirms that the protein has a globular structure with *R*_g_ = 25 Å. The behavior of the dimensionless Kratky plot (Supplementary Figure S7B) also confirms a similarity of the complex FdxE−CYP143 to a compact globular structure in solution. The plot has a maximum value of 1.120 at *qR*_g_ = 1.786, which is very close to the properties of the peak in the case of ideally globular particles (1.104 at *qR*_g_ = √3) (Burger et al., 2016). A peak shift toward higher *qR*_g_ values could indicate partial disorder or an elongated protein shape (Ryzhykau et al., 2021a; Ryzhykau et al., 2021b), but this was not observed in our data.

The distance distribution function *P*(*r*) (Supplementary Figure S7C) was calculated using the GNOM program. Experimental MW was calculated from the Porod volume and as *V*_c_^2^/123.1 *R*_g_ (Rambo and Tainer, 2013). The CORAL program (Petoukhov et al., 2012) was used to perform SAXS-based modeling of the residues of random loops missed in the crystal structure of the fused complex FdxE−CYP143.

We used the EOM program (Tria et al., 2015) from the ATSAS online web platform to fit SAXS experimental data with an ensemble of proteins with variable positions of CYP143 and FdxE. The pool of the structures generated by the EOM corresponds to the distribution of *R*_g_ from 23 Å to 45 Å. However, the experimental *R*_g_ = 25 Å (Supplementary Table S1; Guinier plot is shown in Supplementary Figure S7A) is close to the left border of the pool distribution. The distribution of *R*_g_ for models selected by the genetic algorithm (Supplementary Figure S7B) has a maximum at ∼25 Å and is not symmetrical due to an elongated right-side wing.

The other details of SAXS measurements and data treatment are given in Supplementary Table S1 [prepared in accordance with Trewhella et al. (2017)] along with the data processing results. SAXS data were deposited with SASBDB (http://sasbdb.org) (Kikhney et al., 2020) with accession code SASDPL2.

## Data Availability

The datasets presented in this study can be found in online repositories. The names of the repository/repositories and accession number(s) can be found at: http://www.wwpdb.org/, with accession numbers: 8AMP, 8AMO, 8AMQ.
