# Supplementary material for: Structural insights into 3Fe–4S ferredoxins diversity in M. tuberculosis highlighted by a first redox complex with P450
Source: Front Mol Biosci. 2023 Jan 9;9:1100032. doi: 10.3389/fmolb.2022.1100032 (PMC9868604; doi:10.3389/fmolb.2022.1100032)
Supplement: Supplementary file 1 [file Table1.docx]

## Supplementary materials:

**Fig. S1**. The geometry of [3Fe-4S] cluster (the cluster plus the 3 cysteine Sy ligands) (A) and values of individual bond lengths and angles for Fdx and FdxE and other structurally studied ferredoxins (B).

A


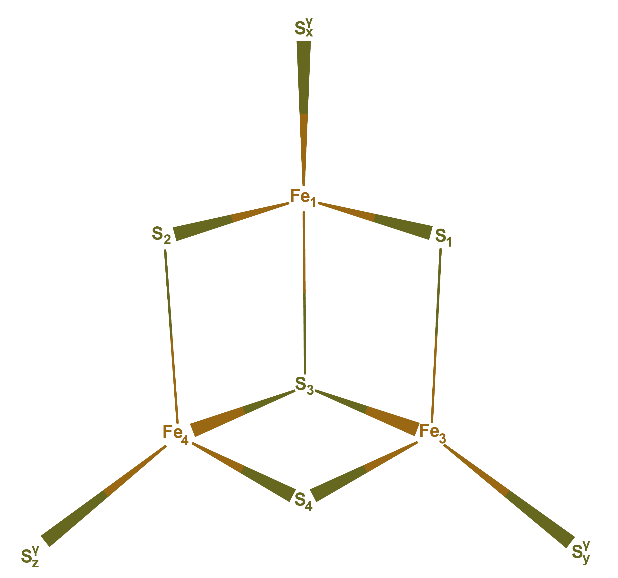


B

| PDB ID | Fe_1_-$S_{x}^{\gamma}$ | Fe_3_-$S_{y}^{\gamma}$ | Fe_4_-$S_{z}^{\gamma}$ | Fe_1_-S_1_ | Fe_1_-S_2_ | Fe_1_-S_3_ | Fe_3_-S_1_ | Fe_3_-S_3_ | Fe_3_-S_4_ | Fe_4_-S_2_ | Fe_4_-S_3_ | Fe_4_-S_4_ |
| --- | --- | --- | --- | --- | --- | --- | --- | --- | --- | --- | --- | --- |
| Fdx | 2.32 | 2.32 | 2.31 | 2.25 | 2.25 | 2.25 | 2.26 | 2.25 | 2.25 | 2.26 | 2.26 | 2.25 |
| FdxE | 2.32 | 2.35 | 2.34 | 2.27 | 2.30 | 2.25 | 2.27 | 2.29 | 2.29 | 2.27 | 2.29 | 2.29 |
| 1FXD | 2.29 | 2.28 | 2.22 | 2.23 | 2.33 | 2.32 | 2.26 | 2.32 | 2.27 | 2.29 | 2.31 | 2.22 |
| 1SJ1_a_ | 2.28 | 2.32 | 2.23 | 2.17 | 2.32 | 2.33 | 2.25 | 2.31 | 2.26 | 2.34 | 2.38 | 2.24 |
| 1SJ1_b_ | 2.27 | 2.32 | 2.28 | 2.23 | 2.32 | 2.35 | 2.33 | 2.36 | 2.23 | 2.32 | 2.31 | 2.32 |
| 4ID8 | 2.31 | 2.25 | 2.19 | 2.21 | 2.22 | 2.19 | 2.21 | 2.14 | 2.24 | 2.27 | 2.16 | 2.21 |
| 4OV1 | 2.39 | 2.30 | 2.25 | 2.21 | 2.19 | 2.23 | 2.22 | 2.20 | 2.20 | 2.23 | 2.21 | 2.21 |

| PDB ID | $S_{x}^{\gamma}$-Fe_1_-S_1_ | $S_{x}^{\gamma}$-Fe_1_-S_2_ | $S_{x}^{\gamma}$-Fe_1_-S_3_ | $S_{y}^{\gamma}$-Fe_3_-S_1_ | $S_{y}^{\gamma}$-Fe_3_-S_3_ | $S_{y}^{\gamma}$-Fe_3_-S_4_ | $S_{z}^{\gamma}$-Fe_4_-S_2_ | $S_{z}^{\gamma}$-Fe_4_-S_3_ | $S_{z}^{\gamma}$-Fe_4_-S_4_ |
| --- | --- | --- | --- | --- | --- | --- | --- | --- | --- |
| Fdx | 101.8 | 114.8 | 116.5 | 104.9 | 120.1 | 110.8 | 117.5 | 102.8 | 116.9 |
| FdxE | 104.9 | 109.7 | 120.4 | 110.3 | 119.3 | 105.5 | 108.4 | 109.1 | 111.7 |
| 1FXD | 106.0 | 114.0 | 118.8 | 110.5 | 116.4 | 116.0 | 110.4 | 110.5 | 121.4 |
| 1SJ1_a_ | 104.7 | 112.9 | 115.3 | 109.9 | 116.6 | 111.3 | 109.8 | 112.1 | 117.6 |
| 1SJ1_b_ | 104.2 | 114.0 | 115.9 | 107.9 | 116.1 | 113.0 | 111.0 | 111.6 | 115.6 |
| 4ID8 | 104.4 | 114.6 | 115.6 | 104.0 | 119.5 | 112.9 | 102.8 | 116.8 | 116.8 |
| 4OV1 | 111.7 | 112.4 | 110.3 | 103.7 | 122.5 | 111.7 | 105.3 | 112.4 | 117.7 |

| PDB ID | S_1_-Fe_1_-S_2_ | S_1_-Fe_1_-S_3_ | S_2_-Fe_1_-S_3_ | S_1_-Fe_3_-S_3_ | S_1_-Fe_3_-S_4_ | S_3_-Fe_3_-S_4_ | S_2_-Fe_4_-S_3_ | S_2_-Fe_4_-S_4_ | S_3_-Fe_4_-S_4_ |
| --- | --- | --- | --- | --- | --- | --- | --- | --- | --- |
| Fdx | 111.1 | 107.0 | 105.5 | 106.8 | 108.4 | 105.4 | 105.2 | 107.5 | 105.4 |
| FdxE | 113.7 | 103.1 | 105.2 | 101.8 | 114.3 | 106.0 | 104.8 | 116.5 | 105.9 |
| 1FXD | 109.2 | 105.1 | 103.3 | 104.2 | 107.2 | 101.3 | 104.8 | 105.0 | 103.4 |
| 1SJ1_a_ | 111.2 | 105.3 | 107.3 | 103.4 | 112.0 | 103.3 | 105.0 | 109.8 | 101.5 |
| 1SJ1_b_ | 109.6 | 106.7 | 106.1 | 103.5 | 111.7 | 104.4 | 107.4 | 107.6 | 103.1 |
| 4ID8 | 112.2 | 103.8 | 105.8 | 105.4 | 108.5 | 105.8 | 104.8 | 108.4 | 106.3 |
| 4OV1 | 119.3 | 101.2 | 100.3 | 101.6 | 114.1 | 103.2 | 99.9 | 117.6 | 102.7 |

| PDB ID | Fe_1_-S_1_- Fe_3_ | Fe_1_-S_3_- Fe_3_ | Fe_3_-S_3_- Fe_4_ | Fe_3_-S_4_- Fe_4_ | Fe_4_-S_2_- Fe_1_ | Fe_4_-S_3_- Fe_1_ |
| --- | --- | --- | --- | --- | --- | --- |
| Fdx | 69.5 | 69.6 | 71.6 | 71.7 | 70.9 | 71.0 |
| FdxE | 73.1 | 73.2 | 69.9 | 69.9 | 70.3 | 70.8 |
| 1FXD | 74.0 | 71.0 | 73.9 | 76.7 | 73.4 | 72.9 |
| 1SJ1_a_ | 73.6 | 69.7 | 71.9 | 75.4 | 71.1 | 70.3 |
| 1SJ1_b_ | 72.3 | 69.8 | 71.3 | 73.5 | 70.6 | 70.2 |
| 4ID8 | 70.8 | 72.7 | 72.7 | 70.0 | 69.9 | 72.5 |
| 4OV1 | 74.7 | 74.6 | 73.8 | 73.9 | 74.8 | 74.5 |

**Fig. S2.** Species name and protein ID (in parenthesis) for ferredoxin sequences in Fig.2.

**mav_1** **-** M.avium (WP_062890415.1); **mma_1** **-** M.marinum (WP_117428654.1; **msm_1** **-** M.smegmatis (WP_003896201.1); **msp_1** – Mycobacterium sp. (WP_009953936.1; **afe_1** –Acidimicrobium ferrooxidans (WP_015798421.1); **aro_1** – Actinospica robiniae (WP_034260932.1); **aau_1** –Arthrobacter aurescens (WP_011777263.1); **cac_1** – Catenulispora acidiphila (WP_015795994.1); **fsp_1** - Frankia_sp. (WP_020464245.1); **faf_1** – Mycobacterium sp. (WP_011724409.1); **nha_1-** Nitrobacter hamburgensis (WP_011511137.1); **nsp_1a** **-** Nitrobacter_sp._Nb311A (WP_009800054.1); **nsp_1b** - Nocardioides_sp. (WP_011757263.1); **nar_1** – Novosphingobium aromaticivorans (WP_011906924.1); **pae_1** – Pseudomonas aeruginosa (WP_121351230.1); **reu_1** - Ralstonia eutropha (WP_136227872.1); **ret_1** – Rhizobium etli (AAM54837.2); **rop_1** – Rhodococcus opacus (WP_015890824.1); **rpa_1** – Rhodopseudomonas palustris (WP_011157460.1); **swi_1** **-** Sphingomonas wittichii (WP_011952583.1); **sco_1** – Streptomyces coelicolor (AGO88622.1); **tfu_1** – Thermobifida fusca (WP_011291910.1); **tcu_1** **-** Thermomonospora curvata (WP_012851129.1); **mab_2a** - M.abscessus (WP_005059330.1); **mav_2a** **-** M.avium (WP_003875758.1); **mma_2** **-** M.ulcerans Agy99 (ABL03175.1); **mtb_2** **-** M.tuberculosis T85 (EFD76427.1); **mva_2** **-** Mycolicibacterium sp. (WP_011782303.1); **mab_2b** **-** Mycobacteroides sp. (WP_180748986.1); **mav_2b** **-** M.avium (WP_224188525.1); **mav_2c** **-** M.avium (WP_003873007.1); **msm_2** **-** M.smegmatis (WP_011730111.1); **msp_2** – Mycolicibacterium monacense (WP_011768425.1); **mul_2** **-** M.marinum (WP_094360996.1); **afe_2** – Acidimicrobium ferrooxidans (WP_015798014.1); **fsp_2a** – Frankia sp. (WP_011435700.1); **fsp_2b** **-** Frankia_sp. (WP_071060280.1); **gbr_2** - Gordonia_bronchialis (WP_012835523.1); **hoc_2** –Haliangium ochraceum (WP_012827286.1); **kfl_2** – Kribbella flavida (WP_012921518.1); **kra_2** – Ktedonobacter racemifer (WP_007922270.1); **mca_2** - Methylococcus capsulatus (WP_010961920.1); **mxa_2** **-** Myxococcus xanthus (DK 1622_ABF90123.1); **nfa_2** – Nocardia sp. (WP_011207065.1); **nsp_2** – Nocardioides sp. (WP_011756255.1); **rer_2** – Rhodococcus erythropolis (WP_073511998.1); **rop_2** – Rhodococcus opacus (WP_015889158.1); **str_2 –** Salinispora tropica (WP_012014024.1); **sav_2** – Streptomyces sp. (WP_010982020.1); **sro_2** – Streptosporangium sp. (WP_012895195.1)

**Fig. S3.** Gene context for Rv076c (A) and Rv1786 (B). Genes colored according to their homology group; non-related genes are in grey. COGs - cluster of orthologous groups. The gene context was generated using MicrobesOnline^21^.

A B


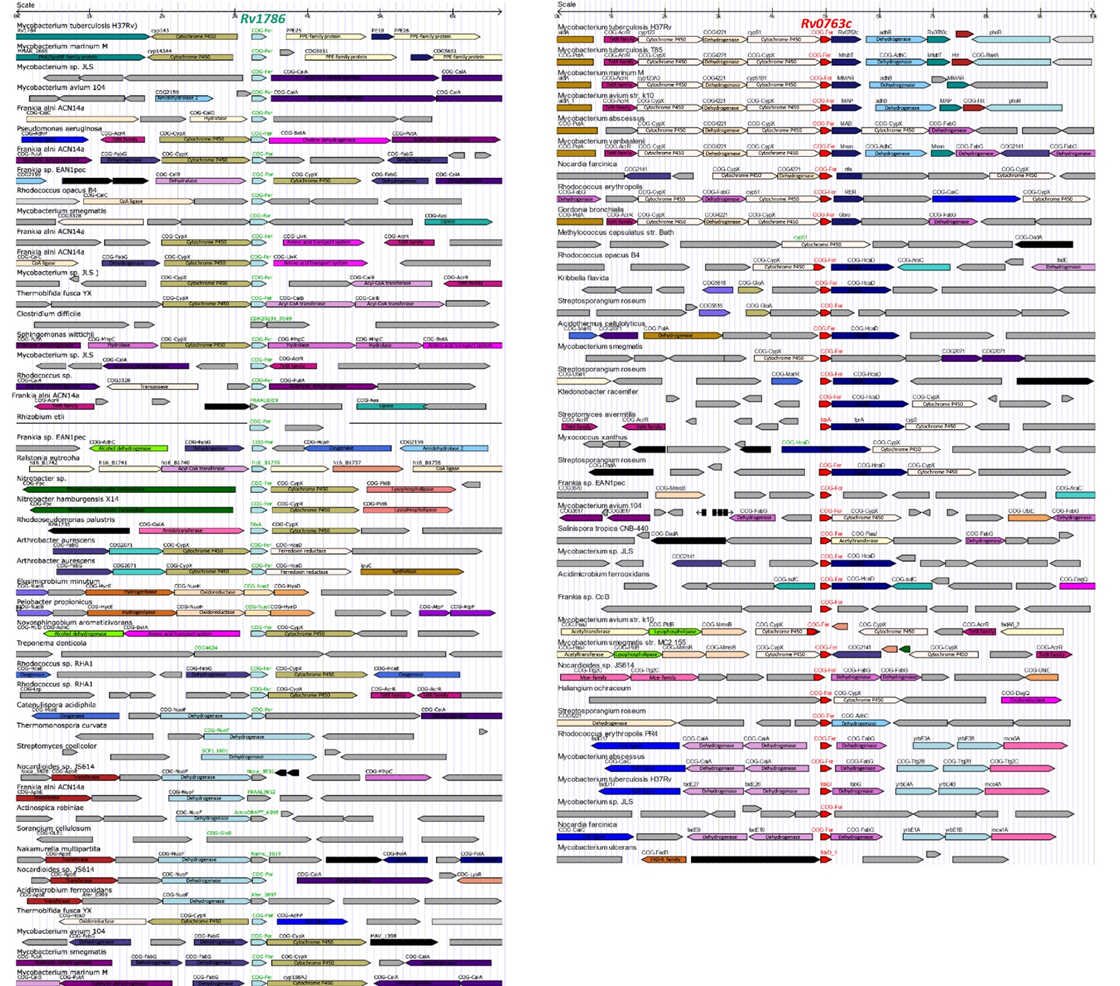


**Fig. S4**. Typical sensorgrams (in red) and their fitting curves (in black) of interactions between biotinylated CYP143 immobilized on SA chip and FdxE in PBS buffer at 25°C. FdxE was injected in follow concentrations: 1 – 10 nM, 2 – 25 nM, 3 – 50 nM, 4 – 100 nM.

**
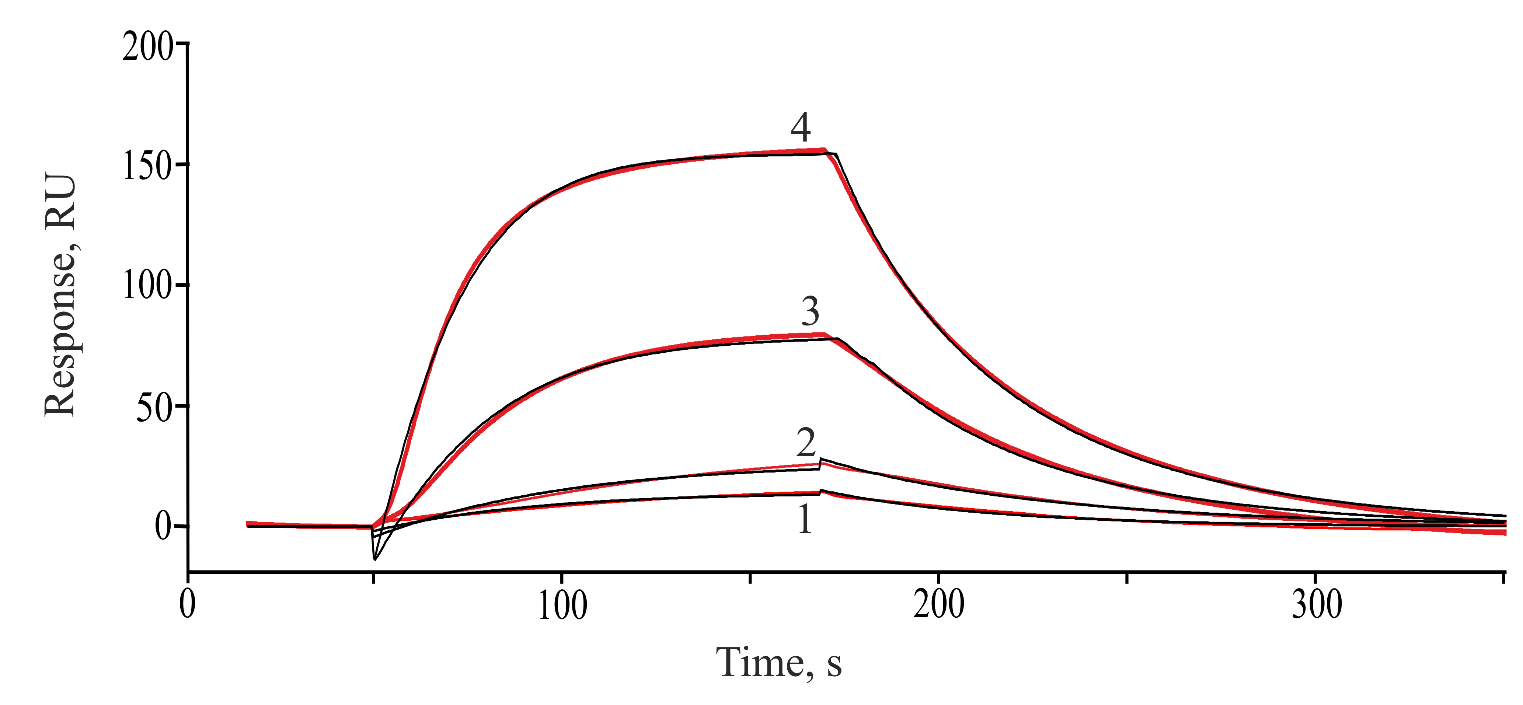
**

**Fig. S5.** The electrostatic surface potentials of the interaction faces of CYP143 (A) and FdxE (B). Negatively and positively charged surface areas are coloured red and blue, respectively. Residues that are involved in the interactions are labeled.

**
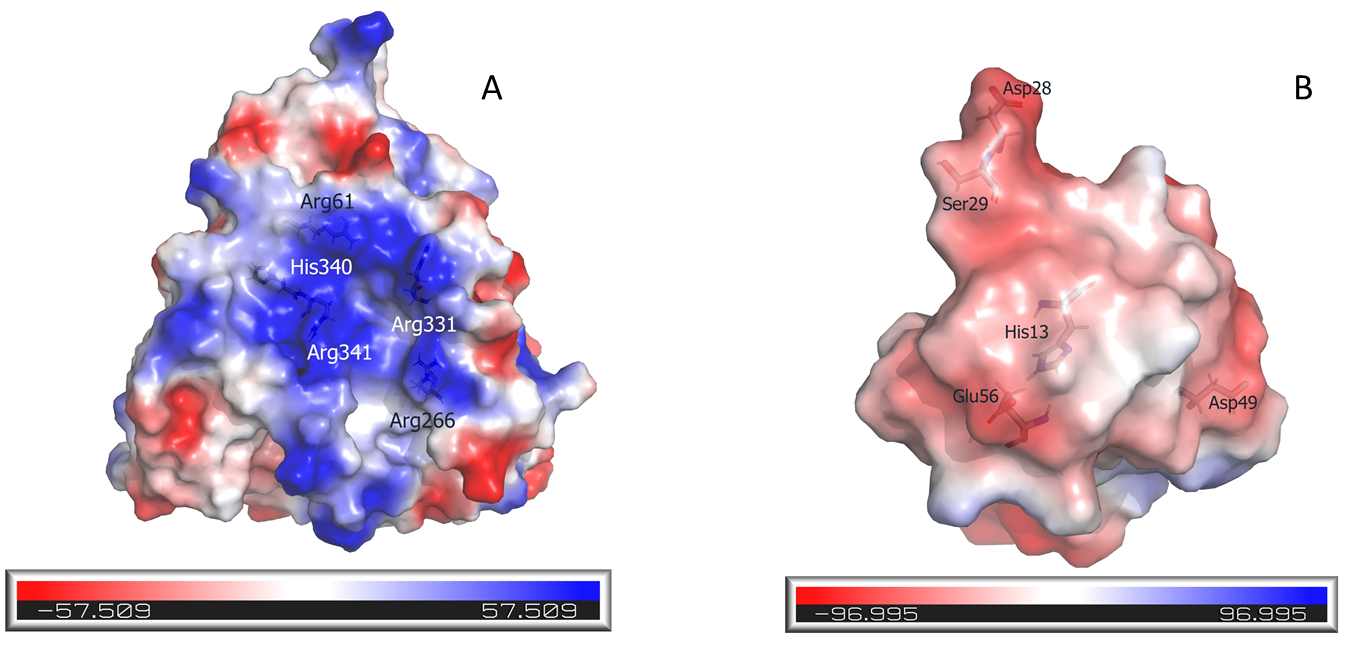
**

**Fig. S6. Fusion FdxE−CYP143 complex obtained with CORAL and EOM.** **A –** Comparison of the models of CORAL model and EOM models obtained with equal weights. The models aligned to the same position of CYP143. **B** – Fusion FdxE−CYP143 complex obtained in EOM with adjustable weights of models. The electrostatic surface potentials are shown for CYP143. Fdx models are shown as cartoons. Fxd in stereospecific complex is colored green. EOM models are colored yellow, pink and orange. Numbers represent occupancies of models.


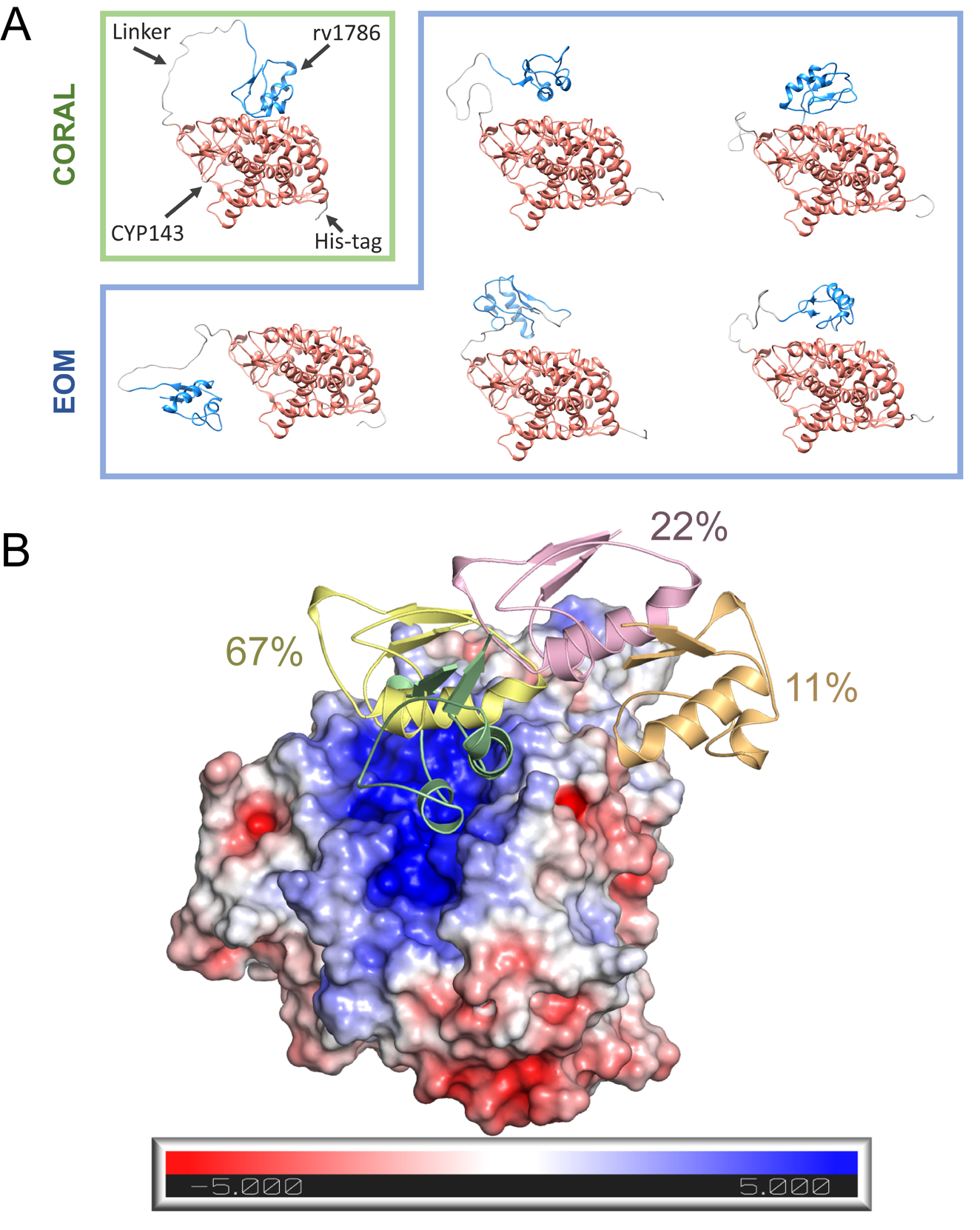


**Fig. S7. Analysis of SAXS data for the** **FdxE****−CYP143 complex. A** – Guinier approximation. **B** – normalized Kratky plot (dashed lines are drawn at *qR*_g_ = $\sqrt{3}$ and (*qR*_g_)^2^ *I*(*q*) / *I*(0) = 1.104). C – pair distance distribution function *P*(*r*).

**Fig. S8.** Summary of the Rv0763c and Rv1786 gene expression. Constructed using <http://www.microbesonline.org/>


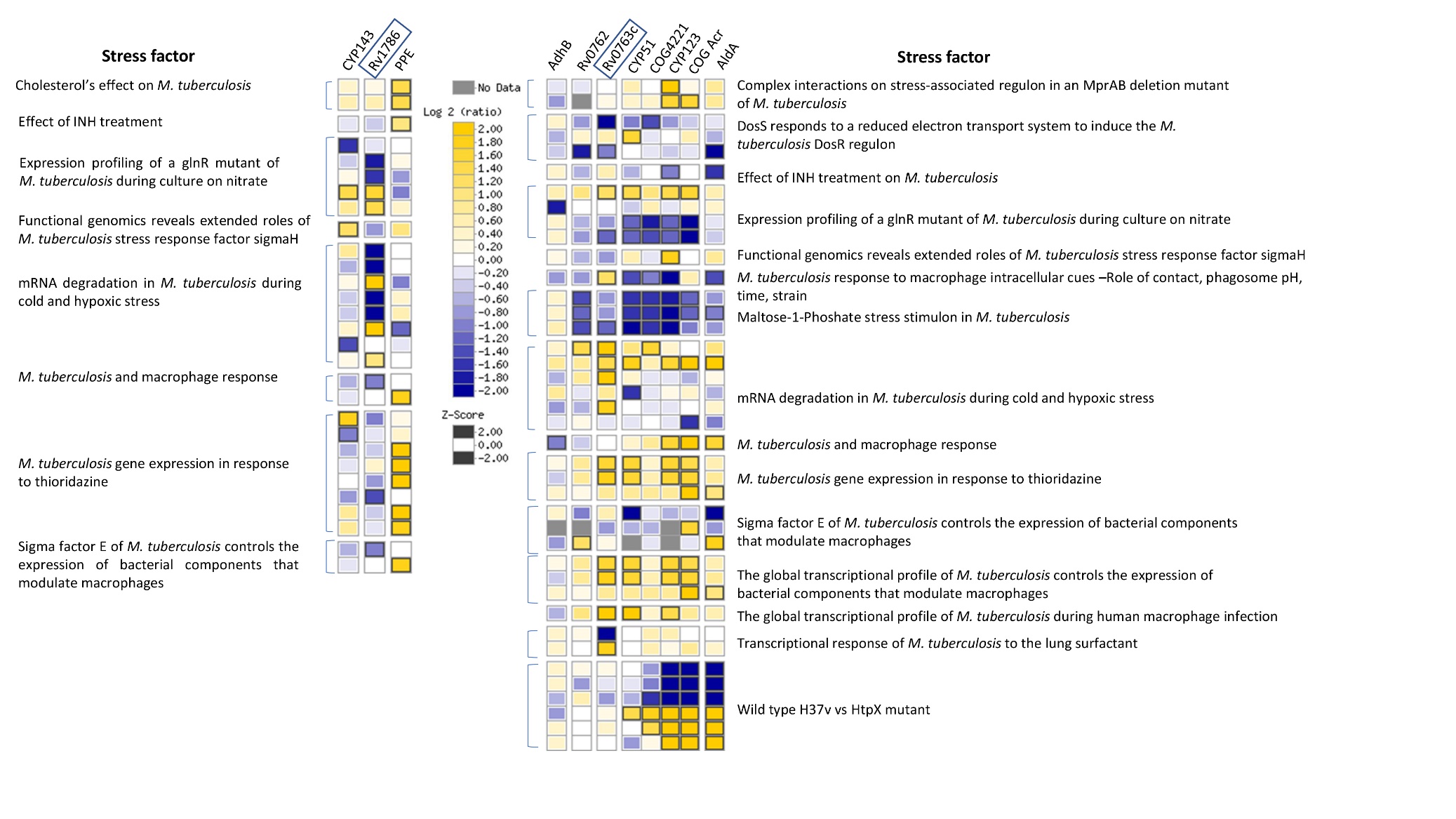


**Fig. S9.** UV-Vis spectra of FdxE (**A**) recorded in 30 mM Tris-HCl, 200 mM NaCl; Fdx (**B**) recorded in 30 mM Tris-HCl, 200 mM NaCl, 20 % glycerol; CYP143 (**C**) recorded in 50 mM Tris-HCl, 300 mM NaCl, 15 % glycerol and FdxE**−**CYP143 (**D**) recorded in 50 mM Tris-HCl, 300 mM NaCl, 15 % glycerol, 1 mM TCEP by using spectrophotometr NanoDrop™ 2000. CO-difference spectra of CYP143 (**C**, **insert**) and FdxE**−**CYP143 (**D**, **insert**) are recorded in 50 mM Potassium-phosphate buffer, pH 7.4 using dual-beam spectrophotometr Cary UV-Vis-NIR 5000 by adding sodium dithionite.

**
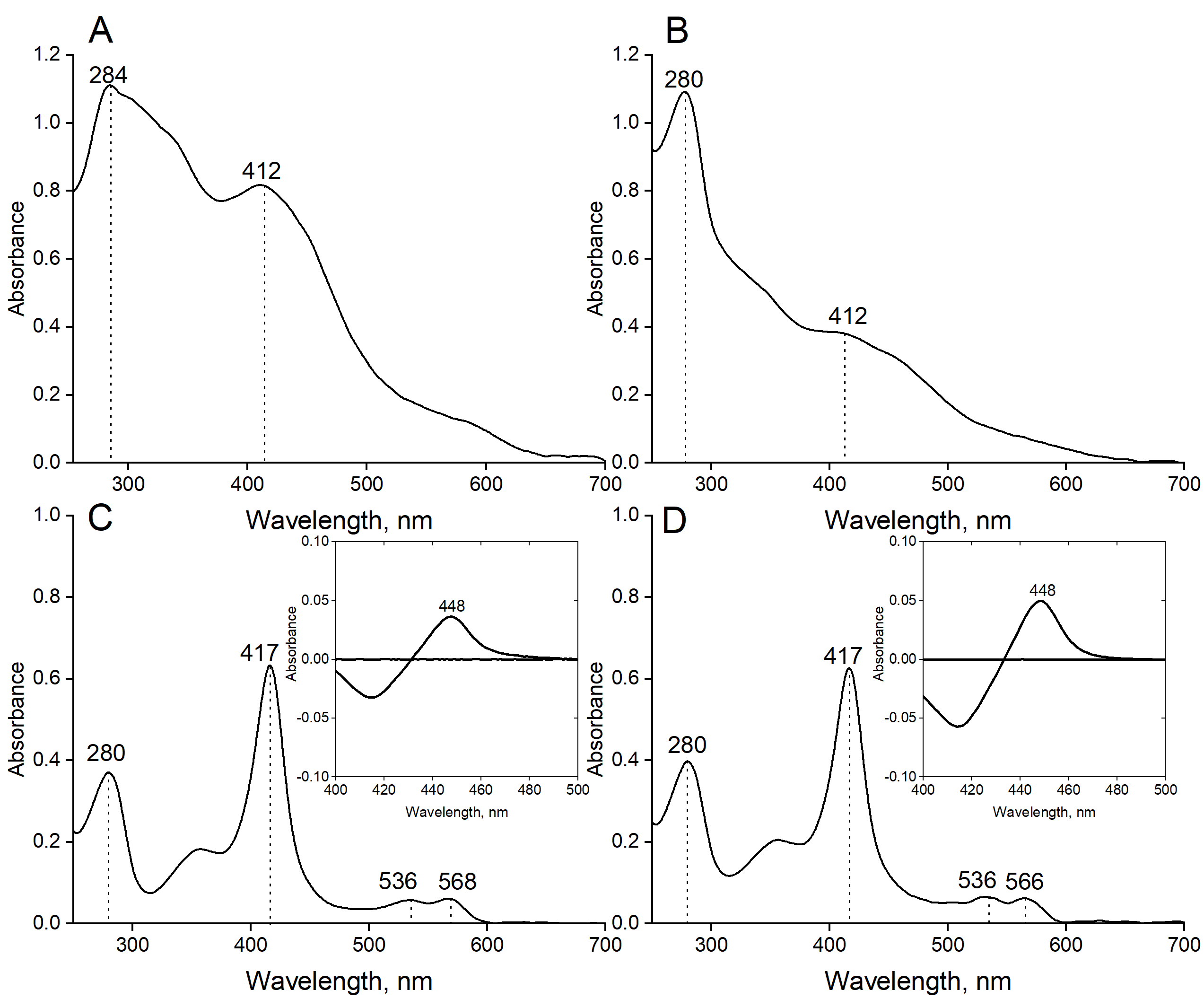
**

**Fig. S10.** 2Fo-Fc and anomalous difference Fourier maps on cofactors. **A, B –** 2Fo-Fc (2.5σ) and anomalous (5.3σ) maps for CYP143, **C, D –** 2Fo-Fc (3σ) and anomalous (3σ) for Fdx, E, F – 2Fo-Fc (7σ) and anomalous (4σ) for Fe-S cluster in FdxE-CYP143 complex, **G, H –** 2Fo-Fc (2.5σ) and anomalous (4σ) for heme in FdxE-CYP143 complex.

**
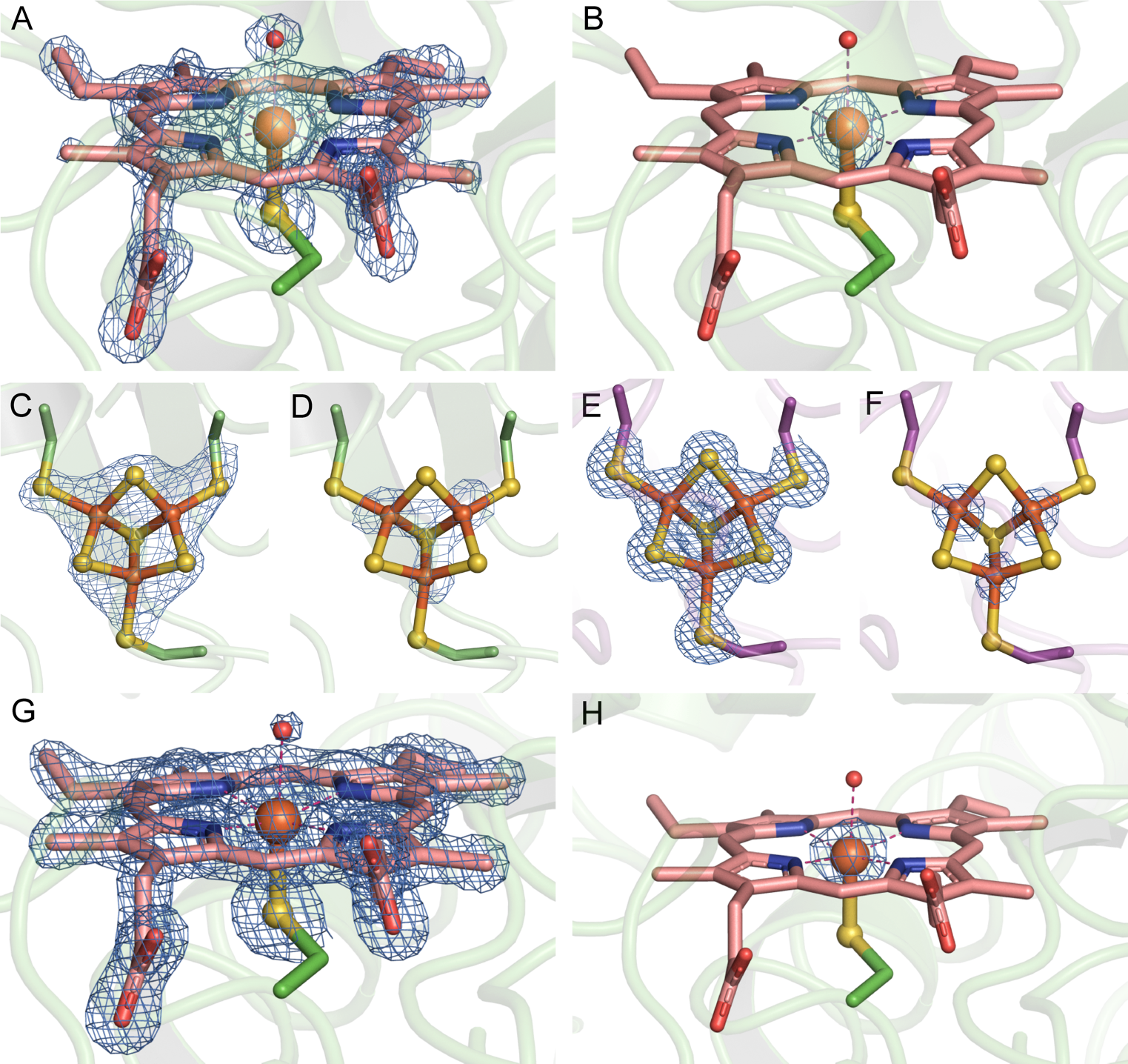
**

**Table S1.** MX data collection and refinement statistics

| Structure | CYP143 | | Fdx | | FdxE–CYP143 | |
| --- | --- | --- | --- | --- | --- | --- |
| PDB ID | 8AMO | | 8AMP | | 8AMQ | |
| **Data collection** | | | | | | |
|  | XSCALE | Staraniso | XSCALE | Staraniso | XSCALE | Staraniso |
| Beamline | ESRF ID23-1 | | ESRF ID30A1 | | ESRF ID30B | |
| Wavelength, Å | 0.972 | | 0.966 | | 0.976 | |
| Space group | P 1 | | H 32 | | P 1 | |
| Unit cell | 42.21, 48.53, 54.15, 111.65, 99.50, 109.34 | | 46.96, 46.96, 162.67, 90, 90, 120 | | 53.20, 54.35, 69.04, 67.71, 77.21, 61.63 | |
| Resolution range (Å)* | 27.78    - 1.40 (1.45    - 1.40) | | 28.76    - 2.00 (2.05    - 2.00) | | 45.32   - 1.60 (1.67    - 1.60) | |
| Resolution limits (Å) | 1.4 | 1.36, 1.60, 1.39 | 2.0 | 1.65, 1.65, 2.57 | 1.6 | 1.55, 1.57, 1.90 |
| No. of total reflections | 469759 (33483) | 401442 (19716) | 46885 (3417) | 46875 (2491) | 274784 (17758) | 232292 (11410) |
| No. of unique reflections | 62494 (4500) | 53501 (2475) | 4958 (346) | 5093 (392) | 78791 (5469) | 66216 (3311) |
| Multiplicity | 7.5 (7.4) | 7.5 (7.4) | 9.5 (9.9) | 9.2 (6.4) | 3.5 (3.4) | 3.5 (3.4) |
| Completeness |  | |  | |  | |
| spherical (%) | 88.8 (86.6) | 76.3 (35.5) | 99.9 (100.0) | 60.6 (17.2) | 94.8 (89.0) | 79.8 (32.7) |
| ellipsoidal (%) | - | 84.6 (71.5) | - | 91.5 (58.3) | - | 91.2 (72.5) |
| Mean I/sigma(I) | 14.0 (0.2) | 15.7 (0.8) | 11.0 (0.9) | 11.0 (0.4) | 4.7 (0.3) | 5.2 (0.8) |
| R-pim | 0.025 (3.771) | 0.023 (1.167) | 0.032 (0.880) | 0.034 (1.768) | 0.078 (1.638) | 0.081 (0.997) |
| CC1/2 | 1.000 (0.243) | 0.999 (0.329) | 0.998 (0.526) | 0.998 (0.127) | 0.994 (0.12) | 0.992 (0.249) |
| **Refinement** | | | | | | |
| Resolution range (Å) | 27.78 – 1.40 | | 28.76 – 2.00 | | 45.32 – 1.60 | |
| Reflections used in refinement | 53378 | | 4119 | | 66205 | |
| Reflections used for R-free | 2654 (5%) | | 416 (10%) | | 3304 (5%) | |
| R-work/R-free | 0.1684/0.1980 | | 0.2465/0.2859 | | 0.1773/0.2057 | |
| No. of non-hydrogen atoms | 3895 | | 487 | | 4479 | |
| macromolecules | 3304 | | 474 | | 3928 | |
| heme | 43 | | - | | 43 | |
| [3Fe-4S] | - | | 7 | | 7 | |
| solvent | 548 | | 6 | | 501 | |
| No. of protein residues | 385 | | 65 | | 464 | |
| RMS |  | |  | |  | |
| bonds (Å) | 0.006 | | 0.005 | | 0.011 | |
| angles (̊) | 0.90 | | 1.02 | | 1.37 | |
| Ramachandran favored (%) | 98.69 | | 100.00 | | 98.25 | |
| Ramachandran outliers (%) | 0.00 | | 0.00 | | 0.00 | |
| Average B-factor | 23.86 | | 56.80 | | 29.95 | |
| macromolecules | 22.65 | | 57.15 | | 29.30 | |
| heme | 15.36 | | - | | 15.82 | |
| [3Fe-4S] | - | | 54.86 | | 20.90 | |
| solvent | 31.83 | | 31.20 | | 36.39 | |
| No. of TLS groups | 3 | | 1 | | 4 | |

*Statistics for the highest-resolution shell are shown in parentheses

**Table S2.** SAXS experimental details and data evaluation summary.

| ***(a)* Sample details** | | | | |
| --- | --- | --- | --- | --- |
|  | CYP143-Rv1786 | | | |
| Description of sequence | His-tagged fused complex of cytochrome P450 143 (UniProt ID: P9WPL3) and ferredoxin Rv1786 (UniProt ID: O53937) from *Mycobacterium tuberculosis* | | | |
| Extinction coefficient ε (A280, 0.1% cm^-1^) **^1^** | 0.890 | | | |
| Partial specific volume ν (cm^3^ g^-1^) **^1^** | 0.729 | | | |
| Mean solute and solvent SLD (10^-6^ Å^-2^) **^1^** | 12.50, 9.75 | | | |
| Mean scattering contrast Δρ (10^-6^ Å^-2^) **^1^** | 2.75 | | | |
| Molecular mass (kDa) **^1^** | 53.561 | | | |
| Sample concentration (mg ml^-1^) | 2.2 | | | |
| Solvent composition | 300 mM NaCl, 50 mM Tris/TrisHCl (pH 7.4), 10% glycerol | | | |
| **(*b*) SAS data collection parameters** | | | | |
| Instrument | ESRF BM29 | | | |
| Wavelength (Å) | 0.9918 | | | |
| Beam geometry (size, sample-to-detector distance) | 700 × 700 µm^2^, 2.864 m | | | |
| Sample configuration | 1.8 mm-diameter quartz capillary | | | |
| *q*-measurement range (Å^-1^) | 0.004 – 0.495 | | | |
| Absolute scaling method | Comparison with scattering from pure H_2_O | | | |
| Basis for normalization to constant counts | To transmitted intensity by direct beam counter | | | |
| Exposure time, number of exposures | 2 frames/sec, 16 frames | | | |
| Sample configuration including path length and flow rate | Sample was exposed to X-rays while flowing through the 1.8 mm-diameter quartz capillary. | | | |
| Sample temperature (°C) | 20 | | | |
| **(*c*) Software employed for SAS data reduction, analysis and interpretation** | | | | |
| SAS data averaging and subtraction | PRIMUS from ATSAS 2.8.4 | | | |
| Calculation of ε from sequence and $\underline{\nu}$ values from chemical composition | Peptide Property Calculator:  <http://biotools.nubic.northwestern.edu/proteincalc.html> | | | |
| Calculation of $\underline{\rho}$ values from chemical composition | SLD calculator: <http://www.ncnr.nist.gov/resources/activation/> | | | |
| Guinier, *P*(*r*) | GNOM 5.0 from ATSAS 3.0.5 | | | |
| Atomic structure modelling | CORAL and EOM from ATSAS 3.0.1 (ATSAS online) | | | |
| Molecular graphics | UCSF Chimera 1.16 | | | |
| **(*d*) Structural parameters** | | | | |
| Guinier analysis |  | | | |
| *I*(0) (cm^-1^) | 0.0812 ± 0.0002 | | | |
| *R*_g_ (Å) | 25.0 ± 0.12 | | | |
| *q*-range (Å^-1^) (*qR*_g_ range) | 0.0236 – 0.0518 (0.59 – 1.29) | | | |
| *P*(*r*) analysis |  |  |  |  |
| *I*(0) (cm^-1^) | 0.0818 ± 0.0002 | | | |
| *R*_g_ (Å) | 25.38 ± 0.11 | | | |
| *d*_max_ (Å) | 90 | | | |
| *q*-range (Å^-1^) | 0.0236 – 0.3528 | | | |
| *q*_min_ *d*_max_ / π | 0.676 | | | |
| Total quality estimate (GNOM) | 0.915 | | | |
| Volume (*V*_P_) (Å^3^) | 85035 | | | |
| Experimental MW from *V*_P_, kDa | 62.0 | | | |
| Experimental MW = *V*_c_^2^ / 123.1 *R*_g_, kDa **^2^** | 53 (± 10%) | | | |
| ***(e)* Atomistic modelling** | | | | |
| Method | SAXS-based rigid body modeling of complexes (CORAL) | | | |
| *q*-range for fitting | 0.0236 – 0.3528 | | | |
| Symmetry assumptions | P1 | | | |
| Background subtraction (cm^-1^) | 0.0007848 | | | |
| χ^2^ value | 1.411 | | | |
| Method | Ensemble Optimization Method (EOM) [with equal weights] | | | |
| *q*-range for fitting | 0.0236 – 0.3528 | | | |
| Symmetry assumptions | P1 | | | |
| Background subtraction (cm^-1^) | 0.000 | | | |
| χ^2^ value | 1.157 | | | |
| *R*_g_ values (Å), *d*_max_ values (Å), and weights for  multi-state model | 24.14, 84.90, ~0.20 (1/5)  27.32, 97.72, ~0.20 (1/5)  24.98, 89.97, ~0.20 (1/5)  24.34, 80.67, ~0.20 (1/5)  24.92, 83.70, ~0.20 (1/5) | | | |
| Final ensemble *R*_g_ (Å) and *d*_max_ (Å) | 25.14, 87.39 | | | |
| Method | EOM [with adjustable weights] | | | |
| *q*-range for fitting | 0.0236 – 0.3528 | | | |
| Symmetry assumptions | P1 | | | |
| Background subtraction (cm^-1^) | 0.000 | | | |
| χ^2^ value | 1.139 | | | |
| *R*_g_ values (Å), *d*_max_ values (Å), and weights for  multi-state model | 25.48, 82.69, ~0.11 (1/9)  24.66, 91.93, ~0.67 (6/9)  26.77, 104.82, ~0.22 (2/9) | | | |
| Final ensemble *R*_g_ (Å) and *d*_max_ (Å) | 25.22, 93.77 | | | |
| ***(f)* Data and model deposition IDs** | | | | |
|  | CYP143-rv1786  SASDPL2 | | | |

**^1^** These values are calculated without taking into account ligands: heme for CYP143 and 3Fe-4S for FdxE.

**^2^** MW is calculated as *V*_c_^2^ / 123.1 *R*_g_ according to^79^.
